# Prognostic value of cervical length for spontaneous preterm birth in asymptomatic women with twin pregnancy: meta-analysis of individual participant data

**DOI:** 10.1136/bmjmed-2024-000877

**Published:** 2025-04-16

**Authors:** Kelly Margaret Hughes, Mason Aberoumand, Anna Lene Seidler, Phoebe Swan, Mona Aboulghar, Maria de Lourdes Brizot, Clifton Brock, Marta Benito Vielba, Nathan Fox, Cynthia Gyamfi-Bannerman, Lindsay Kindinger, Giorgio Pagani, Alfredo Perales Marin, Viola Seravalli, Mariarosaria Di Tommaso, Omer Weitzner, Katharina Worda, Lukas Staub, Shaun Brennecke, Shakila Thangaratinam, Ben W Mol, Rui Wang

**Affiliations:** 1 Department of Obstetrics and Gynaecology, Monash University, Clayton, Victoria, Australia; 2 NHMRC Clinical Trial Centre, University of Sydney, Sydney, New South Wales, Australia; 3 Department of Child and Adolescent Psychiatry, Rostock University Medical Center, Rostock, Germany; 4 Department of Obstetrics and Gynaecology, Cairo University, Giza, Egypt; 5 Disciplina de Obstetricia, Departamento de Obstetricia e Ginecologia, Faculdade de Medicina, Universidade de Sao Paulo, Sao Paulo, Brazil; 6 Midwest Fetal Care Center, Children’s Minnesota - Minneapolis, Minneapolis, Minnesota, USA; 7 Department of Obstetrics and Gynecology, Miguel Servet University Hospital, Zaragoza, Spain; 8 Department of Obstetrics, Gynecology, and Reproductive Science, Icahn School of Medicine at Mount Sinai, New York, New York, USA; 9 Departments of Obstetrics, Gynecology, and Reproductive Sciences, University of California San Diego, La Jolla, California, USA; 10 King Edward Memorial Hospital for Women Perth, Subiaco, Western Australia, Australia; 11 Maternal Fetal Medicine Unit, Department of Obstetrics and Gynecology, ASST Papa Giovanni XXIII, Bergamo, Italy; 12 Department of Obstetrics, La Fe University and Polytechnic Hospital, Valencia, Spain; 13 Department of Health Sciences, University of Florence, Firenze, Italy; 14 Department of Obstetrics and Gynaecology, University of Toronto, Toronto, Ontario, Canada; 15 Department of Obstetrics and Gynecology, Medical University of Vienna, Wien, Austria; 16 Department of Obstetrics and Gynaecology, University of Melbourne Faculty of Medicine Dentistry and Health Sciences, Melbourne, Victoria, Australia; 17 Pregnancy Research Centre, The Royal Women’s Hospital, Parkville, Victoria, Australia; 18 University of Birmingham Institute of Metabolism and Systems Research, Birmingham, UK; 19 Department of Obstetrics and Gynaecology, Monash University Faculty of Medicine Nursing and Health Sciences, Clayton, Victoria, Australia; 20 Department of Obstetrics and Gynaecology, University of Aberdeen, Aberdeen, UK

**Keywords:** Pregnancy complications, Ultrasonography, Diagnostic imaging, Prenatal care

## Abstract

**Objective:**

To quantify the prognostic value of mid-trimester cervical length for spontaneous preterm birth in asymptomatic women with twin pregnancy and to assess whether other factors may modify any association.

**Designs:**

A two stage meta-analysis of individual participant data in a Cox proportional hazard model was performed using cervical length as a continuous variable.

**Data sources:**

Medline, Embase, Cochrane, and LILACS, among others, were searched to identify eligible studies; the search was from 1 January 2000 to 30 September 2020. Risk of bias was assessed with the QUIPS tool. Studies were from eight countries between 2001 and 2018.

**Eligibility criteria:**

Individual participant data were sought for eligible studies that reported mid-trimester (defined between 16 and 26 weeks) transvaginal sonographic cervical length and also gestational age at birth in asymptomatic women with twin pregnancy. The primary outcome was spontaneous preterm birth before 37 weeks.

**Results:**

Among 29 eligible studies, authors of 17 studies provided individual participant data for 6437 women with a twin pregnancy (69.1% of individual participant data). Mean cervical length measurement was 39 mm (SD=9, range 1-74 mm). 2889 women (44.9%) delivered before 37 weeks' gestation, and 934 (14.9%) delivered before 34 weeks. Each 1 mm increase in cervical length was associated with a 4.0% reduction in the rate of spontaneous preterm birth before 37 weeks (hazard ratio 0.96 (95% confidence interval 0.95 to 0.97)), and a 6.8% reduction in the rate of spontaneous preterm birth before 34 weeks' gestation (0.93 (0.92 to 0.95)). The prognostic value remained stable in models adjusting for different sets of variables.

**Conclusion:**

The prognostic value of cervical length for spontaneous preterm birth in twin pregnancy is on a continuous scale. No specific cervical length has been identified that can reliably predict or exclude all spontaneous preterm births.

**Study registration:**

CRD42020146987.

**WHAT IS ALREADY KNOWN ON THIS TOPIC**

**WHAT THIS STUDY ADDS**

- No single cut-off value of cervical length was reported to differentiate between high risk and low or normal risk pregnancy

**HOW THIS STUDY MIGHT AFFECT RESEARCH, PRACTICE, OR POLICY**

- The findings recommend against the use of a single cut-off value (traditionally 25 mm) to differentiate between high-risk and low or normal-risk pregnancy

## Introduction

 Preterm birth, defined as birth before 37 weeks' gestation, is the leading cause of perinatal and early childhood mortality worldwide. Preterm birth is a risk factor for death from other causes and is associated with short and long term morbidity.^1^ Preterm birth is more frequent in women with a twin pregnancy, with reported rates as high as 65%.^2^ While preterm birth may be indicated due to maternal or fetal condition (iatrogenic), spontaneous preterm birth (SPTB) is potentially predictable and preventable. SPTB has a range of proposed biological causes; twin pregnancy is an independent risk factor, possibly due to uterine overdistension.^3^ Changing demographics, including increasing maternal age and use of assisted reproductive technologies, have seen an increase in the number of twin pregnancies, and therefore a larger population at risk.^4^

The length of the uterine cervix, measured via transvaginal ultrasound in the second trimester in asymptomatic women, has been proposed as an important prognostic factor for SPTB.^5^ We have published evidence, however, that existing aggregate data meta-analyses have fundamental limitations that necessitate caution in applying their conclusions.^6^ Briefly, aggregate data were not consistently available due to limited reporting. The use of arbitrary dichotomisation with a broad range of cut-offs for the prognostic factors and primary outcome restricted the ability to synthesise aggregate data from primary studies. Additionally, statistical analyses used diagnostic tests rather than a contemporary prognostic research framework.^6^

An individual participant data meta-analysis describes the collation of raw, row-by-row datasets. This method allows for inclusion of all available data, analysis on a continuous scale, and application of consistent outcome definitions,^7^ to address the limitations of previous aggregate data meta-analyses.^8 9^ The objective of this meta-analysis was to quantify the prognostic value of sonographically determined cervical length in mid-trimester (also known as second trimester) for SPTB in asymptomatic women with twin pregnancy.

## Methods

### Protocol and registration

The research protocol was registered with PROSPERO (www.crd.york.ac.uk, CRD42020146987). The systematic review underpinning this meta-analysis has been published previously.^9^ These results are reported according to the PRISMA-individual participant data checklist (online supplemental appendix 1). All analyses were prespecified and described in detail in the statistical analysis plan (online supplemental appendix 2).

### Eligibility criteria

Studies meeting the following criteria were eligible for inclusion. Participants were asymptomatic women with twin pregnancy and with no more than 20% of each cohort receiving treatment to reduce the risk of spontaneous preterm birth (progesterone, cerclage, pessary). Indexed prognostic factor: transvaginal ultrasound performed with a widely accepted technique (ie, as described by Iams et al^10^ or Fetal Medicine Foundation).^11^ Outcomes from studies needed to include both gestational age at delivery and classification as spontaneous or iatrogenic preterm birth available in individual participant data. For timing, pregnancy was dated by standard methods (early dating ultrasound or last menstrual period), and gestational age at transvaginal length measurement occurred between 16 and 26 weeks. Only cohort studies or placebo or untreated groups from randomised controlled trials were included, in which data were collected after the year 2000 (due to limited availability of older data), and n ≥100.

### Patient and public involvement

Patients or the public were not involved in the design, conduct, reporting, or dissemination plans of our research because the meta-analysis was based on previously collected datasets.

### Search criteria

An information specialist helped design the search strategy. We searched Medline, Embase, CINAHL, LILACS, Database of Abstracts of Reviews of Effects (DARE), Cochrane database, JBI Database of Systematic Reviews, ClinicalTrials.gov, and Google Scholar, from 1 January 2000 to 30 September 2020. No language restriction was applied. Citation tracking was performed. We also consulted experts in the field.

The following keywords were searched: cervix uteri, uterine cervical incompetence, cervical length measurement, ultrasonography, prenatal, (cervix or cervical) length, (pre-term or preterm or premature), (delivery or birth or labour or labour), abortion, spontaneous. The full strategy is available in the online supplemental appendix 3.

Title and abstract screening were performed by two authors (KH, RW). Full-text review was completed by two reviewers (KH, PS, or RW), with conflicts resolved by a third reviewer (RW or BWM).

### Data collection processes

Contact details were sought for the corresponding or first author, or, where that was unavailable or outdated, any listed author. We sent an introductory email to at least one author of each citation and sent at least two further emails (with alternate addresses tried where available) if we received no response. Where contact details were outdated, we attempted to locate the current institution of the author or enquired via other research contacts in a similar geographical area.

If an author responded positively, we arranged an online meeting or a data sharing agreement, or both. De-identified data were transferred by secure file transfer platform (our recommended mode of sharing) or email, and uploaded to a secure university server. Data queries were resolved by email. Individual participant data was sought for all eligible studies where an author was contactable, including those published only as conference abstracts.

### Data items

We sought entire datasets that included, at a minimum, cervical length, gestational age at measurement of cervical length, and gestational age at birth. The main prognostic factor was cervical length, measured in millimetres as close to 20 weeks' gestation. In cases where only a range for gestational age at the time of measurement of cervical length for the entire individual study was provided rather than individual level data, the mean of that range was imputed as the gestational age. In cases where multiple cervical length measurements were available per participant, we selected the measurement closest to 20 weeks' gestation due to it coinciding with the usual morphology ultrasound.

We also aimed to collect other prognostic factors, including but not limited to maternal age, body mass index, nulliparity, history of preterm births, and chorionicity. Prognostic factor selection among prespecified clinically meaningful prognostic factors was guided by availability of data across studies (at least 50% of data points) and descriptive analyses of the association between prognostic factor and outcome.

For the outcome variables, the primary outcome was time to spontaneous preterm birth (time-to-event outcome). This event was estimated by censoring the outcome of gestational age at 37 weeks, only including spontaneous preterm births, and by calculating survival models. Preterm prelabour rupture of membranes was counted as a spontaneous preterm birth even if delivery was due to induction of labour or if it occurred after 37 weeks' gestation; the rationale being that the process was pathological and commenced prematurely, even if the birth was completed at term. We treated any medically indicated preterm births as censored observations.

The secondary outcome was time to any type of preterm birth (spontaneous or iatrogenic, from any cause) by censoring the outcome of gestational age at 37 or 34 weeks to include any preterm birth and calculating survival models.

Prior to conducting the analyses, all individual participant data were checked with respect to range, internal consistency, missing or extreme values, errors, and consistency with published reports. Concerns about data inconsistency and integrity were solved by contacting the leading author of the original publication. If concerns could not be addressed adequately, we excluded the dataset in question.

### Risk of bias assessment in individual studies

The quality in prognosis studies (QUIPS) tool^12^ was used to assess risk of bias in six domains: study participation, study attrition, prognostic factor measurement, outcome measurement, adjustment for other prognostic factors and statistical analysis, and reporting. For domains on adjustment for other prognostic factors and statistical analysis and reporting, the assessments were based on the individual participant data. Studies that were only published in abstract form were not assessed for risk of bias because information was insufficient. Two reviewers (KH, MA) assessed the included studies, and conflicts were resolved by consensus or a third reviewer (RW).

### Synthesis methods

SAS software version 9.4 (SAS Institute Inc.) was used to compile the datasets from the participating studies, harmonising and processing was performed with R software.^13^

For descriptive analyses, we plotted and tabulated the following descriptive analyses in each study: characteristics of participants; distribution of measured cervical length; associations between measured cervical length and gestational age at cervical length measurement; associations between other included prognostic factors and cervical length.

We performed a two stage meta-analysis with R, using individual participant data. The two stage approach was selected because most included studies were of sufficient size for the assumptions to be appropriate. The first stage comprised building regression models for each study using all the explanatory variables available in each study. We built Cox proportional hazard models, producing hazard ratios (HRs) with 95% confidence intervals (CIs).

We planned to build multiple models, as follows:

1. The primary model was a basic model including only cervical length and gestational age at measurement as prognostic factors (model 1);
2. A model with only key demographic prognostic factors (ie, mother's age and nulliparity) and cervical length (model 2); and
3. A model with all potential prognostic factors (model 3a), and a model with all potential prognostic factors, only including studies that utilised all factors (model 3b)

In the second stage, the results of the study level regression models were meta-analysed in a random-effects model using the inverse variance method. Forest plots were produced for all models. The percentage of the total variability in study estimates due to between-study variance was determined using the I^2^ statistic and measured with $\tau^{2}$ . The 95% prediction interval (PrI) was calculated using a previously described inverse variance method.^14^ Small study effects were investigated with contour enhanced funnel plots and Egger's regression test. Cervical length was modelled as continuous and a linear association on the log-hazard scale was assumed.

If data were systematically missing at study level, the two stage meta-analysis described here allowed for outcome estimates using all the available variables in each study. Complete case analysis was performed.

### Additional analyses

We used penalised splines to investigate the non-linear relations between cervical length and time to SPTB for each trial. The number of knots was four based on the recommendation from Harrell,^7^ which were then pooled together in a two stage meta-analysis.

Subgroup analyses were planned to detect whether cervical length prognosticates SPTB differently across different populations. For this purpose, interaction terms were introduced into the primary model for the primary outcome of SPTB.

Prespecified subgroup analyses were: history of cervical excisional surgery; uterine anomaly; previous preterm birth; previous spontaneous preterm birth; nulliparous women; and multiparous women with a previous term birth.

We planned the following sensitivity analyses for the primary model:

Exclude women who receive treatment to reduce the risk of preterm birth (cerclage, progesterone, pessary), time-to-event; exclude studies in which any woman received treatment; spontaneous preterm birth (<37 weeks), binary outcome; spontaneous preterm birth (<34 weeks), binary outcome; spontaneous preterm birth (<30 weeks), binary outcome; exclude studies with an overall high risk of bias.

We also built logistic regression models to consider the outcomes as binary outcomes and produced odds ratios with 95% CIs.

### Patient and public involvement

Patients or the public were not involved in the design, conduct, reporting, or dissemination plans of our research because our study used previously collected datasets. We have shared preliminary results of this study with clinicians and researchers via the Perinatal Society of Australia and New Zealand conference, and would hope that these results will continue to be shared by clinicians to women with twin pregnancies.

## Results

### Search results

**Figure 1:**
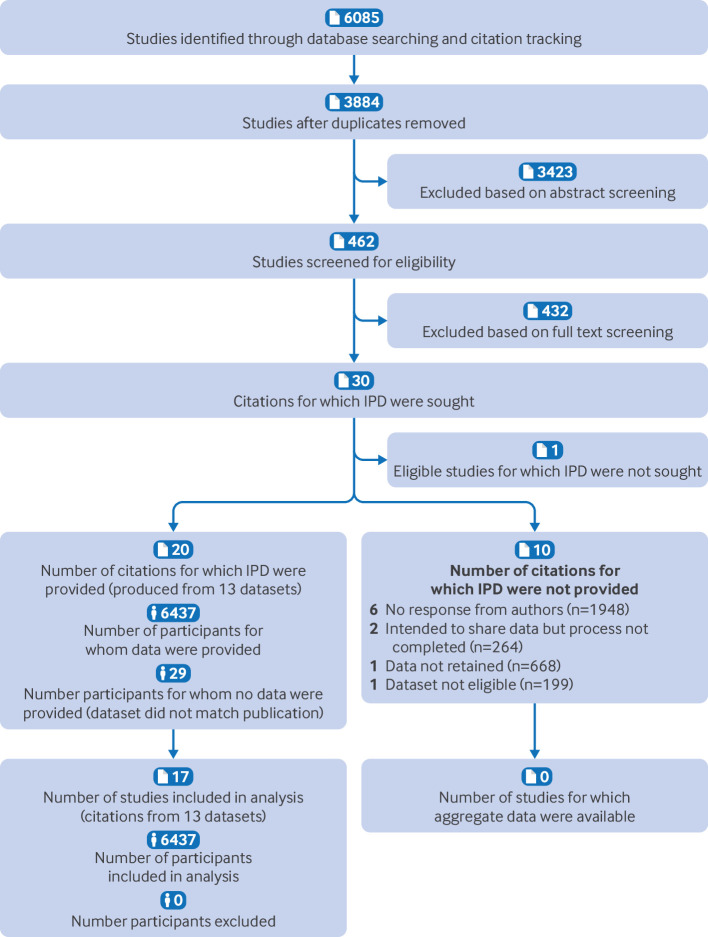
PRISMA flow chart. One eligible study was excluded before IPD were sought because a main author had already declined to participate. IPD-MA IPD=individual participant data

**Figure 2:**
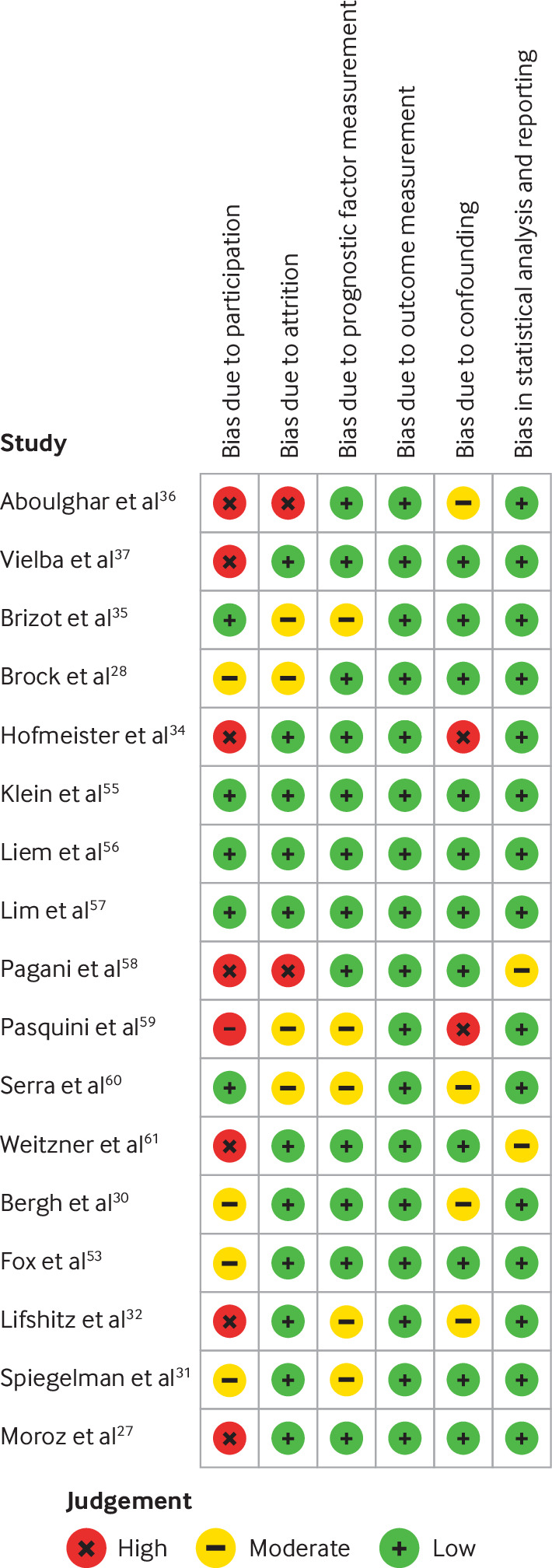
Risk of bias assessment that used the quality in prognosis studies (QUIPS) tool

The literature search produced 6085 citations. After duplicates were excluded, 3884 abstracts were screened. We reviewed 462 full text articles, and 30 eligible citations remained (figure 1). Six authors could not be contacted,^15^,^20^ a further two intended to share data but did not complete the process,^21 22^ and one author had discarded their study data.^23^ Of the 20 eligible citations with data available, three were conference citations that could not undergo risk of bias assessment (therefore 17 full text articles underwent risk of bias assessment; figure 2).^24^,^26^ Gyamfi-Bannerman and colleagues contributed the latter citations and two more,^27 28^ which were all produced from one dataset (papers assessed separately for risk of bias, listed in figure 2 as first authors Moroz and Brock). Fox and colleagues contributed four citations produced from their dataset.^29^,^32^ One author offered an additional relevant dataset, used in three citations.^33^,^35^ Therefore, 17 citations resulted in 13 discrete datasets^35 36^ with 6437 pregnancies of a possible 9317 (69.1%) eligible pregnancies with individual participant data. Studies where individual participant data could not be obtained are listed in online supplemental table 1. One author had expanded their dataset after the end date of the literature search and published a study based on these newer data^37^; they provided their larger dataset for inclusion. No other concerns were raised about data integrity.

### Study characteristics

Study characteristics and demographic data are shown in table 1.

**Table 1:** Summaries of included studies

Study	Year	Country	Study design	No. of participants (n=6437)	Mean cervical length, mm (SD)	Mean GA at delivery, weeks (SD)	% SPTB <37 weeks (no. of events) (n=2823)	% SPTB <34 weeks	Demographic data reported
Aboulghar et al^*36*^	2009	Egypt	Non-randomised controlled/comparative trial	193	36 (9)	34.46 (4.07)	74.1 (143)	25.9	None
Benito Vielba et al^37^	2021	Spain	Retrospective cohort	424	39 (7)	36.39 (2.52)	39.9 (169)	10.4	Smoking, white ethnic group, BMI
Brizot et al^35^	2015	Brazil	Randomised controlled trial	318	38 (8)	35.85 (2.75)	54.4 (173)	15.7	Smoking, white ethnic group
Brock et al, Moroz et al^24 27 28 51^	2016, 2017, 2017, 2019	USA	Retrospective cohort	1222	41 (10)	35.37 (2.87)	20.3 (248)	10.6	White ethnic group
Fox et al, Lifshitz et al, Bergh et al, Spiegelman et al^30^ ^31^ 3252,54	2009, 2014, 2015, 2016	USA	Retrospective cohort	1102	39 (9)	35.70 (2.73)	30.9 (340)	12.1	White ethnic group
Hofmeister et al^34^	2010	Brazil	Retrospective cohort	385	38 (9)	35.64 (2.75)	50.6 (195)	13.8	None
Klein et al^55^	2008	Austria	Retrospective cohort	223	36 (8)	35.53 (2.75)	71.3 (159)	14.3	Smoking
Liem et al^56^	2013	Netherlands	Randomised controlled trial	593	44 (9)	35.59 (3.54)	52.8 (313)	18.7	Smoking, white ethnic group
Lim et al^57^	2012	Netherlands	Randomised controlled trial	456	43 (10)	36.11 (3.18)	46.3 (211)	16.0	Smoking, white ethnic group
Pagani et al^58^	2016	Italy	Retrospective cohort	940	37 (8)	35.17 (2.87)	69.6 (654)	21.2	Smoking, white ethnic group, BMI
Pasquini et al^59^	2017	Italy	Retrospective cohort	222	37 (9)	35.84 (2.06)	50.0 (111)	9.0	None
Serra et al^60^	2013	Spain	Randomised controlled trial	256	38 (8)	36.41 (2.69)	11.3^49^	11.3	Smoking
Weitzner et al^61^	2020	Israel	Retrospective cohort	103	38 (8)	36.50 (2.13)	10.7^58^	10.7	None

BMI, body mass index; GA, gestational age; SD, standard deviation; SPTB, spontaneous preterm birth.

The studies were conducted in eight countries between 2001 and 2018. The mean maternal age was 33.5 years (standard deviation (SD) 5.9). 4839 (75.2%) twin pregnancies were dichorionic, 1189 (18.5%) twin pregnancies were monochorionic, and chorionicity was not reported for 409 (6.4%) pregnancies. Monochorionic-monoamniotic pregnancies were excluded from all primary studies.

Mean gestational age at cervical length measurement was 20.54 weeks' gestation (SD 1.73), and mean cervical length was 39 mm (SD 9). 459 women (7.1% of the cohort) received prophylactic treatments (table 1), under 4% for each treatment modality.

Mean gestational age at birth was 35.63 weeks (SD 3.01) (online supplemental figure 1). 2889 women (44.9%) had a SPTB before 37 weeks' gestation, and 934 (14.9%) had a SPTB before 34 weeks. A positive correlation was noted between gestational age at birth and cervical length (sample correlation estimate 0.25, P<0.001; online supplemental figure 2).

### Risk of bias assessments

Three studies were at low risk of bias across all domains. 12 of 17 studies were at moderate or high risk of bias in the participation domain. Six of 17 studies were at moderate or high risk of bias in the domains for attrition and adjustment for other prognostic factors (figure 2).

### Individual participant data meta-analysis

**Figure 3:**
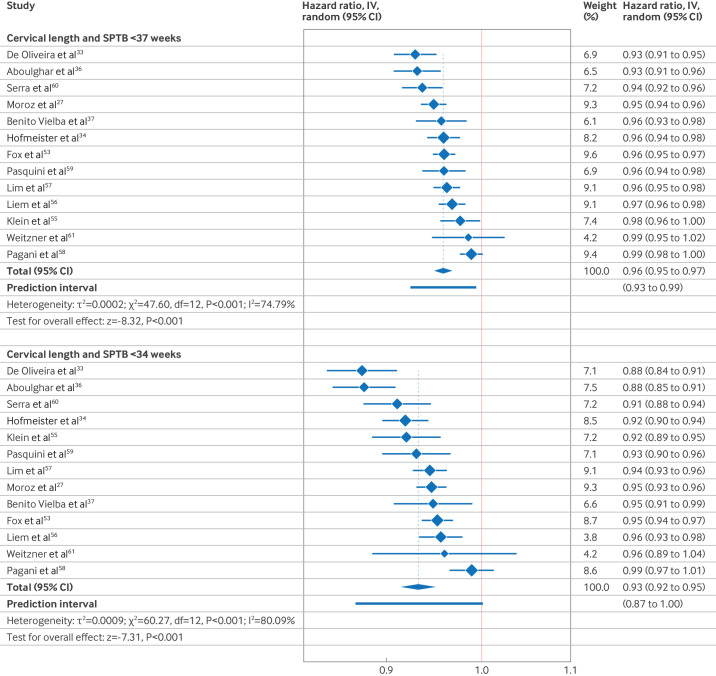
Forest plots of cervical length and SPTB before 37 and 34 weeks' gestation. CI=confidence interval; SPTB=spontaneous preterm birth; IV=instrumental variable

**Figure 4:**
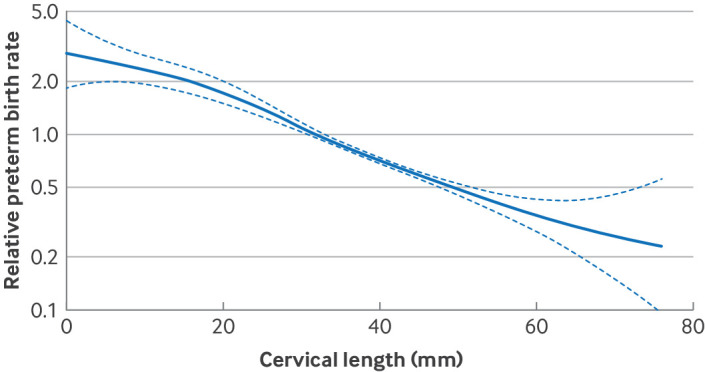
Investigation for non-linear association between cervical length and spontaneous preterm birth

The primary model included only cervical length and gestational age at measurement of cervical length (model 1). The individual participant data meta-analysis showed that each additional millimetre of cervical length was associated with a 4.0% decreased rate of SPTB before 37 weeks' gestation (13 studies, 6437 participants, hazard ratio (HR) 0.96, 95% CI 0.95 to 0.97, PrI 0.93 to 0.99; figure 3) and a 6.8% decreased rate of SPTB less than 34 weeks' gestation (13 studies, 6437 participants, HR 0.93, 95% CI 0.92 to 0.95, PrI 0.87 to 0.99; figure 3). When investigating the non-linear relation between cervical length and SPTB, the spline is very close to a linear association (figure 4). Between-study heterogeneity was low (τ^2^=0.0002 for <37 weeks, τ^2^=0.0009 for <34 weeks) and most of the total variability in study estimates was due to between-study variance (I^2^=74.8% for <37 weeks, I^2^=80.1% for <34 weeks). The contour enhanced funnel plot showed no evidence of small study effects.

A similar prognostic value was observed when adding maternal age and nulliparous status into the model (model 2, nine studies, 4660 participants, HR 0.96 95% CI 0.95 to 0.97, 95% PrI 0.95 to 0.97, τ^2^<0.0001) or further adding gestational age at cervical length measurement, maternal age, nulliparity, and chorionicity into the model (model 3, 13 studies, 6437 participants, HR 0.96, 95% CI 0.95 to 0.97, 95% PrI 0.93 to 1.0,τ^2^=0.0002). When limiting the individual participant data meta-analysis to studies with all the covariates in model 3, the prognostic value remained similar (model 3b, seven studies, 4438 participants, HR 0.96 (CI 0.94 to 0.98), PrI 0.938 to 0.981).

The secondary outcome of cervical length's association with any preterm birth produced an HR of 0.96 (CI 0.954 to 0.972), PrI 0.930 to 0.997) (online supplemental figure 3).

We were unable to perform the planned subgroup analyses because necessary variables were not collected in the primary studies. Sensitivity analyses are presented in online supplemental table 2.

## Discussion

### Summary of evidence

69.1% of published individual participant data (total number of pregnancies) on mid-trimester cervical length in asymptomatic women with twin pregnancy was obtained, with a combined dataset of 6437 pregnancies.

The primary model assessing the association between cervical length nearest to 20 weeks' gestation and rate of SPTB performed at least as well as multivariable models. The data showed a 6.8% reduction in the rate of SPTB prior to 34 weeks' gestation for every millimetre increase in cervical length, and a 4.0% reduction in the rate of SPTB before 37 weeks' gestation.

### Strengths and limitations

Strengths of this analysis include a thorough literature review designed by an information specialist and extensive attempts to obtain individual participant data, including those from grey literature. We have identified cervical length as a prognostic factor (rather than as a diagnostic test, which has been the case in most previous literature)^9^ and used contemporary statistical methods for prognostic factor research. We have analysed cervical length and gestational age as continuous variables (as well as applying clinically important cutoffs for gestational age) to avoid the data loss associated with dichotomisation.^38 39^ We acknowledge use of restricted maximum likelihood is more common with the Hartung-Knapp-Sidik-Jonkman adjustment approach; however, in our case, the findings and interpretation are similar (online supplemental table 3).

Additionally, we acknowledge that uncomplicated dichorionic diamniotic twin births are planned around 37+0 to 37+6 weeks’ gestation, which is still relevant to the standard definition of preterm birth, but that timing of birth, even in uncomplicated monochorionic twin pregnancies, is earlier than this timeframe.^40^

A further limitation is the proportion of individual participant data that was unavailable (approximately 30%). We found that several authors had left the institutions at which they had previously published, their contact details were no longer valid, or they were unable to be found at other institutions. Additionally, the data collection phase occurred during the initial years of the Covid-19 pandemic, which meant that authors may have been restricted in their activities or directly affected by disease.

Despite the τ^2^ values suggesting that between-study heterogeneity is low, the rates of SPTB varied widely among datasets (range 10.7-74.1%). While the variation in SPTB rates and cervical length between continents is well described,^41^ this variation is unlikely to sufficiently explain the results seen in our analysis. Study source populations differed, which may provide some further explanation. For example, Aboulghar et al studied a population entirely comprising pregnancies that used intracytoplasmic sperm injection,^36^ which would be at higher baseline risk of SPTB than spontaneously conceived twin pregnancies.^42^

The most common study design in this area is a retrospective cohort, which typically introduces selection bias from the outset. Perhaps for the convenience of data collection and statistical analysis, participants with incomplete outcome data are nearly always excluded from the dataset. Therefore, whether a group of women with similar characteristics may have been systematically excluded from each study is not possible to find out. A possible hypothesis is that low socioeconomic status, a known risk factor for SPTB,^43^ is associated with lack of housing security, which may mean women do not deliver at the hospital with which they booked. This may have some impact on the association between cervical length and SPTB, but we are unable to investigate this further due to limitations with this dataset.

Similarly, women with iatrogenic preterm birth are frequently excluded from these analyses. We found that cervical length had a similar prognostic value for both SPTB and all preterm birth; however, many datasets excluded women with iatrogenic preterm birth prior to analysis, which would skew this result. We would not expect cervical length to be affected by these conditions. For future studies, we would advocate not excluding these women, but censoring participants in whom preterm birth is indicated for maternal or fetal reasons, and using multiple imputation for missing data. Similarly, our use of complete case analysis may be considered a limitation. While we used the default method of the statistical package, missing data might not have been at random.^44^

Primary studies all excluded monochorionic-monoamniotic twin pregnancies, which is a reasonable strategy given the high rate of complications and preterm birth.^45^ The findings of this analysis, therefore, are unable to provide further risk stratification for these women.

We found the datasets collected to be lacking information on potential cofactors, which limited our ability to explore multivariable prediction models. Even in a large sample of twin pregnancies, which are more likely to be the result of artificial reproductive technology than singletons,^46^ these data were not collected. Other variables, even several that are well known to be associated with SPTB, such as tobacco use and socioeconomic status, were also not reported. We had intended to assess neonatal outcomes; however, these data were not collected in the primary studies. At present, clinicians tend to use ad hoc prediction models generated by adding known historical or demographic risk factors to ultrasound findings to assign levels of risk to a pregnancy; however, this approach is not validated and prediction models have tended to perform poorly.^47^ Our results provide evidence that twin pregnancy confers a high a priori risk of SPTB, and that a shorter cervix is associated with a higher risk. Further analysis of this dataset may allow precise risk estimates at various cervical length cutoffs, however, we have already cautioned extensively against arbitrary dichotomisation.

We would certainly not be the first group to report on the challenges of data collection for individual participant data meta-analysis, but some issues warrant further discussion. Contacting authors several years post-publication via the details provided for journals was a time consuming task as many had moved to other institutions. Data sharing could be more readily facilitated if first authors were encouraged to provide an ongoing email address as a secondary contact. Additionally, the legal aspect of data sharing substantially lengthens the data collection process; in this project, exchanges between representatives of our institution and another took over two years to negotiate mutually satisfactory terms. This process seems to be amplified when different legal jurisdictions are involved, for example, data sharing from an EU country with a non-EU country. This delay substantially impacts the recency of our results and limits our ability to gain a comprehensive combined dataset from available literature because more studies have undoubtedly been published in the duration of these legal negotiations. While we are sympathetic to the intent of legal protections for privacy, we suggest that participants in primary studies have either already prospectively provided consent for data collection for research, or that consent was not required for the collection of deidentified retrospective data. The legal challenges delay research advances and hinder patient care, and we would urge institutions and governing bodies to consider streamlining these processes.

### Clinical and research implications

Our analysis shows that no critical prognostic cervical length is available for SPTB in twin pregnancy because the hazard increases linearly as cervical length decreases, and likewise, that no safe cervical length, at which the possibility of SPTB can be excluded, was defined. Previous research has not consistently shown benefit for prophylactic treatments such as vaginal progesterone and cervical cerclage in twin pregnancy.^48^ As supporting evidence gathers for the treatment of women with a shorter cervix,^49 50^ this information may be used to help determine at what cervical length treatment may be of benefit.

For future research undertaken in this area, we would urge researchers to plan comprehensive prospective data collection in cohorts of women without excluding those who have iatrogenic preterm births.

## Conclusions

Mid-trimester transvaginal cervical length in asymptomatic women with twin pregnancy is associated with the risk of spontaneous preterm birth, but its prognostic capacity is not improved by the addition of select potential co-factors. Every 1 mm increase in cervical length reduces the rate of birth before 34 weeks' gestation by 6.8%, and before 37 weeks by 4.0%.

## Supplementary material

10.1136/bmjmed-2024-000877 online supplemental appendix 1

10.1136/bmjmed-2024-000877 online supplemental appendix 2

10.1136/bmjmed-2024-000877 online supplemental appendix 3

10.1136/bmjmed-2024-000877 online supplemental figure 1

10.1136/bmjmed-2024-000877 online supplemental figure 2

10.1136/bmjmed-2024-000877 online supplemental figure 3

10.1136/bmjmed-2024-000877 online supplemental table 1

10.1136/bmjmed-2024-000877 online supplemental table 2

10.1136/bmjmed-2024-000877 online supplemental table 3

## Data Availability

Data are available upon reasonable request.
