# Supplementary material for: Prognostic value of cervical length for spontaneous preterm birth in asymptomatic women with twin pregnancy: meta-analysis of individual participant data
Source: BMJ Med. 2025 Apr 16;4(1):e000877. doi: 10.1136/bmjmed-2024-000877 (PMC12056617; doi:10.1136/bmjmed-2024-000877)
Supplement: online supplemental appendix 2 [file bmjmed-4-1-s002.pdf]

Appendix 2:

Statistical Analysis Plan

The ability of mid-trimester transvaginal ultrasonographic cervical length to predict spontaneous preterm birth in asymptomatic women with twin pregnancy: an individual participant data meta-analysis

Prepared by: Lukas Staub, Anna Lene Seidler, Kelly Hughes, Rui Wang, Mason Aberoumand

1. Activity Log

1.1. SAP revisions

6 November 2020: First version (V1)
20 November 2020: Second version (V2)
8 December 2020: Third version (V3)
9 December 2020: Fourth version (V4)
18 December 2020: Fifth version (V5)
21 January 2021: Sixth version (V6)
21 January 2021: Eight version (V8)
5 March 2021: Ninth version (V9)
30 July 2021: Tenth version (V10)
16 August 2021: Eleventh version (V11)
07 September 2021: Twelth version (V12)
28 September 2021: Thirteenth version (V13)

2. Study Overview

2.1. Background and introduction

Preterm birth is in quantity and severity the most important issue in obstetric care in the world. In Australia, preterm delivery occurs in approximately 8.6% of all deliveries, while these rates rise to 63% for multiple pregnancies. Preterm delivery can either be iatrogenic (i.e. medically indicated) or spontaneous, with iatrogenic preterm birth being 3.9% and spontaneous onset being 4.7% of all deliveries in 2013.

Spontaneous preterm birth is linked to adverse outcomes, including increased risk of infant mortality and severe morbidity, and is associated with an increased risk of spontaneous

preterm birth in a subsequent pregnancy. Prevention of spontaneous preterm delivery is therefore a major perinatal research priority.

A preventative strategy for spontaneous preterm birth is dependent on the identification of high-risk women and the effectiveness of subsequent treatment. Potential treatments include cerclage, pessary and progestogens, while aspirin (certain) and dietary interventions (likely) are also effective.

The focus of this project is on the identification of high-risk women for spontaneous preterm delivery. Over the last decades, many studies have been performed to address prediction of spontaneous preterm birth. However, coordination of these efforts has been lacking and therefore research resources have been used suboptimally. To allow optimal use of current data, the best type of evidence synthesis is individual participant data (IPD) meta-analysis.

Background, rationale and general methodology of the IPPIC-II project have been documented in the grant proposal.

Previous evidence suggests that mid-trimester transvaginal ultrasonographic cervical length (CL) measurement may be an important predictor of spontaneous preterm birth. This statistical analysis plan sets out the analyses for an IPD meta-analysis assessing the value of mid-trimester transvaginal ultrasonographic CL measurement in predicting preterm birth. The plan proposes a method of quantifying changes in the probability of preterm birth, accounting for mid-trimester cervical length among other potential effects.

These methods have been selected to best allow for the effects to be incorporated into future decision support tools.

2.2. Aims and hypotheses

This study aims to assess whether a transvaginal ultrasonographic cervical length measurement in the second trimester can predict spontaneous preterm birth in asymptomatic women with twin pregnancy, with or without additional risk factors. We will assess women with singleton pregnancy in a separate parallel study.

2.3. Presentation of results

The validity of the conclusions will be discussed with members of the participating studies. The results and insights from this analysis will be published in a peer-reviewed journal, and presented at relevant conferences.

2.4. Variables of interest

2.4.1. Outcomes

Primary outcome

The primary outcome is gestational age in weeks at spontaneous preterm birth (time-to-event outcome), This is calculated by censoring the outcome of gestational age at 37 weeks to only include spontaneous preterm births, and calculating survival models. Studies that also reported medically indicated preterm birth, are treated as censored observation.

Secondary outcomes

We will consider the following secondary outcome:

- Time to any type of preterm birth (spontaneous or iatrogenic, any cause). This is calculated by censoring the outcome of gestational age at 37 weeks to only include any preterm birth, and calculating survival models.

2.4.2. Possible explanatory variables

The main predictor is cervical length (CL) measured in millimetres.

Below is a list of the additional available explanatory variables for the IPD meta-analysis, these were assumed to provide a minimum set of variables that can be used as predictors in this analysis.

Note that most explanatory variables are not available in all studies.

Table1. Predictors of gestational age in the model analysis for multiple pregnancies

Variable name	Description	Values
Age_mother	Mother's age	numeric
Nullipara	Nullipara	0=no; 1=yes
Hx_PTB	History of preterm births	numeric
Chorionicity	Chorionicity in multiple pregnancies	1=monochorionic; 2=dichorionic
CL	Cervical length	[mm]
GA_measurement	Gestational age at CL measurement	[weeks]

Predictor selection among pre-specified clinically meaningful predictors will be guided by:

- Availability of data across studies (only predictors that are available for at least 50% of data points will be included)
- Descriptive analyses of association between predictor and outcome

In cases where multiple CL measurements are available per participant, we will select the measurement closest to 20 weeks gestation due to its proximity to the usual morphology ultrasound.

In addition, only CL measurements taken between gestational week 16 to 24 will be included in the analysis. In cases were only a range for gestational age at the time of measurement of the cervical length is provided for the entire individual study rather than individual level data, the mean of that range will be imputed as the gestational age.

All predictors will be specified before we start building models in our main analysis.

3. Risk of bias assessment

QUIPS will be used to assess risk of bias. Each study will be assessed by two independent reviewers. Conflicts will be resolved in a consensus meeting or by consulting a third reviewer.

4. Statistical Analyses

4.1. Data

This analysis will combine data from the following 17 studies of mid-trimester transvaginal ultrasonographic cervical length measurement:

Table 2. Included studies

Study	Abbreviation	N
Aboulghar et al	Abo	193
Arabin et al	Ara	459
Brizot et al	Bri	318
Fox	Fox	1102
Hofmeister et al	Hof	385
Klein	Kle	223
Liem et al	Lie	593
Lim et al	Lim	463
Serra et al	Ser	256
Sperling et al	Spe	383
Aboulghar	Abo2	199
Weitzner	Wei	103
Pagani	Pag	911
Gyamfi-Bannerman	Gya	1229
Seravalli	Serv	222

4.2. Statistical significance

The statistical significance will be set at α=0.05 throughout the study.

4.3. Analysis methods

All analyses will be performed separately for studies of singleton and those of multiple pregnancies (use variable 'Multiples' to select studies), and these analyses will be published separately. This present statistical analysis plan is for the multiple analyses only.

Prior to conducting the analyses all data will be checked with respect to range, internal consistency, missing or extreme values, errors and consistency with published reports.

For any individual outcome there will be an a-priori decision whether the quality of data is sufficient to allow meaningful analysis. If this is not the case, the analysis will not be conducted, and this will be reported in the publication.

Descriptive analyses

We will plot and tabulate the following analyses in a descriptive way:

- Characteristics of patients included in each study
- Distribution of measured CL in each study
- Relationship between measured CL and gestational age at CL measurement in each study
- Relationships between other included predictors and CL in each study

IPD meta-analysis

The primary outcome of interest for the analysis will be the association between CL measurement and the incidence of preterm birth. We will perform a two-stage IPD meta-analysis.

The first stage will comprise building regression models for each study, using all the explanatory variables available in each study. Correlations between multiples will be accounted for in the analyses. We will build cox proportional hazard models and present hazard ratios with 95% confidence intervals. We will build multiple models, as follows:

1) **Basic model including only cervical length as a predictor (Model 1) – primary model**
2) Model with only key demographic predictors (mother's age, nullipara) and cervical length (Model 2)
3) Model with all predictors in table 1 (Model 3)
4) Model with all predictors in table 1, only including studies that include all predictors (Model 4)

In the second stage, the results of the study-level regression models will be meta-analysed in a random-effects model. Forest plots will be presented for all models, and between-study heterogeneity will be determined using I^2 statistic, between-study heterogeneity variance τ^2 and the prediction interval. Small study effect will be investigated with contour enhanced funnel plots.

We will present cumulative incidence curves to visualise Model 1.

4.4. Subgroup analysis

Individual participant level data allows for great flexibility in conducting the statistical analysis to identify different risk factors that may predispose to spontaneous preterm birth via different mechanisms (which may or may not involve cervical shortening).

Subgroup analyses will be performed to detect if cervical length predicts preterm birth differently across different populations. For this purpose, interaction terms will be introduced into Model 1, for the primary outcome of spontaneous preterm birth.

Pre-specified subgroup analyses include:

- History PT birth
- History of cervical surgery
- Uterine anomaly

4.5. Sensitivity analyses

We will conduct the following sensitivity analyses for Model 1:

- Exclude women that receive treatment for preterm birth (Cerclage, Progesterone, Pessary), time-to-event
- Spontaneous PT birth (<37 weeks), binary outcome
- Spontaneous PT birth (<34 weeks), binary outcome
- Spontaneous PT birth (<30 weeks), binary outcome
- Exclude studies with an overall high risk of bias

We will build logistic regression models for binary outcomes, and present results as odd ratios with 95% confidence intervals. In addition, the non-linear association between cervical length and preterm birth will be investigated with generalised additive models such as splines.

4.6. Missing data

Given the aggregate nature of the data sets it is likely that there will be missing or not applicable variables between the studies. Multiple imputation is very difficult to perform for study-level missing data, instead the two-level meta-analysis described here will allow for outcome estimates using all the available variables in each study.

4.7. Software

SAS version 9.4 will be used to compile the datasets from the participating studies.

All subsequent statistical analyses will be conducted in R version 4.1.0.

5. List of all available variables

**Variable name**	**Description**	**Values**
Study	Study ID	Abbreviations
Multiples	Study of multiple pregnancies	0=no; 1=yes
PID	PatientID within each study	numeric
Age_mother	Mother's age	numeric
Gravidity	Gravidity	numeric
Nullipara	Nullipara	0=no; 1=yes
Chorionicity	Chorionicity in multiple pregnancies	1=monochorionic; 2=dichorionic
IVF	Mode of conception IVF	0=no; 1=yes
CL	Cervical length	[mm]
GA_measurement	Gestational age at CL measurement	[weeks]
Cerclage	Cerclage in present pregnancy	0=no; 1=yes
Progesterone	Progesterone	0=no; 1=yes
Pessary	Pessary	0=no; 1=yes
GA_delivery	Gestational age at delivery	[weeks]
PTB_spontaneous	Spontaneous preterm birth	0=no; 1=yes
PTB_indicated	Preterm birth with clinical indication	0=no; 1=yes
Delivery_mode	Mode of delivery	1=vaginal; 2=Caesarean section
Sex_newborn	Sex of newborn	1=female; 2=male
Birthweight_newborn	Birthweight of newborn	[grams]

6. Appendix

Variable name	Inclusion:	Comment/Reason
Ethnicity	Exclude	Not enough studies. CL may vary across ethnicities but we don't have enough data on this. Maybe able to do

		as Caucasian/non-Caucasian, as the largest datasets have this at minimum
Smoking	Exclude	Not enough studies. Could be considered.
BMI mother	Exclude	Not enough studies. Previous studies find mixed evidence
DM mother	Exclude	Not available in any study
Hypertension mother	Exclude	Not available in any study
HX cervical history	Exclude	Not available in any study
Uterine anomaly	Exclude	Not enough studies
UTI mother	Exclude	Not enough studies
Gravidity	Exclude	Not enough studies, Nullipara already included
HX_term_births	Exclude	Not enough studies
Hx_miscarriage	Exclude	Not enough studies
IVF	Include	Not enough studies, this would be important to look at heterogeneity.
Gestational Diabetes	Exclude	Not enough studies
Diabetes_gravidarum	Exclude	Not enough studies
Cerclage	Exclude	Not enough studies
HELLP	Exclude	Not enough studies, this variable is not well-described enough in the articles to include as a variable
PROM	Exclude	Not enough studies
Progesterone	Exclude	Not enough studies, studies with this item as treatment will be considered for sensitivity analysis

Pessary	Exclude	Not enough studies, studies with this item as treatment will be considered for sensitivity analysis
Mode of delivery	Exclude	Not enough studies
Birthweight	Exclude	Not enough data
Sex	Exclude	Not enough data
