# Supplementary material for: Prognostic value of cervical length for spontaneous preterm birth in asymptomatic women with twin pregnancy: meta-analysis of individual participant data
Source: BMJ Med. 2025 Apr 16;4(1):e000877. doi: 10.1136/bmjmed-2024-000877 (PMC12056617; doi:10.1136/bmjmed-2024-000877)
Supplement: online supplemental appendix 3 [file bmjmed-4-1-s003.pdf]

Appendix 3: literature search strategy (as applied in Ovid Medline)

Search number	Term	Limit
1	"screening to prevent spontaneous preterm birth".mp.	[mp=title, abstract, original title, name of substance word, subject heading word, floating sub-heading word, keyword heading word, organism supplementary concept word, protocol supplementary concept word, rare disease supplementary concept word, unique identifier, synonyms]
2	"length of the cervix and the risk of spontaneous".mp.	[mp=title, abstract, original title, name of substance word, subject heading word, floating sub-heading word, keyword heading word, organism supplementary concept word, protocol supplementary concept word, rare disease supplementary concept word, unique identifier, synonyms]
3	"predictive accuracy of serial transvaginal cervical lengths".mp	. [mp=title, abstract, original title, name of substance word, subject heading word, floating subheading word, keyword heading word, organism supplementary concept word, protocol supplementary concept word, rare disease supplementary concept word, unique identifier, synonyms]
4	((cervix or cervical) adj3 length).mp.	[mp=title, abstract, original title, name of substance word, subject heading word, floating sub-heading word, keyword heading word, organism supplementary concept word, protocol supplementary concept word, rare disease supplementary concept word, unique identifier, synonyms]
5	Cervix Uteri/	None
6	Uterine Cervical Incompetence/	None
7	Cervical Length Measurement/	None
8	Ultrasonography, Prenatal/	None
9	4 or 5 or 6 or 7 or 8	None

10	((pre-term or preterm or premature) adj (delivery or birth or labour or labor)).mp.	[mp=title, abstract, original title, name of substance word, subject heading word, floating sub-heading word, keyword heading word, organism supplementary concept word, protocol supplementary concept word, rare disease supplementary concept word, unique identifier, synonyms]
11	exp Obstetric Labor, Premature/	None
12	abortion, spontaneous/	None
13	10 or 11 or 12	None
14	9 and 13	None
15	4 or 7 or 8	None
16	13 and 15	None
17	(review* or systematic or meta* or umbrella).mp.	[mp=title, abstract, original title, name of substance word, subject heading word, floating sub-heading word, keyword heading word, organism supplementary concept word, protocol supplementary concept word, rare disease supplementary concept word, unique identifier, synonyms]
18	16 and 17	None
19	14 not 16	None
20	1 and 16	None
