# Supplementary figures and images for: Prognostic value of cervical length for spontaneous preterm birth in asymptomatic women with twin pregnancy: meta-analysis of individual participant data
Source: BMJ Med. 2025 Apr 16;4(1):e000877. doi: 10.1136/bmjmed-2024-000877 (PMC12056617; doi:10.1136/bmjmed-2024-000877)

Supplementary figure 1: CL distributions by study

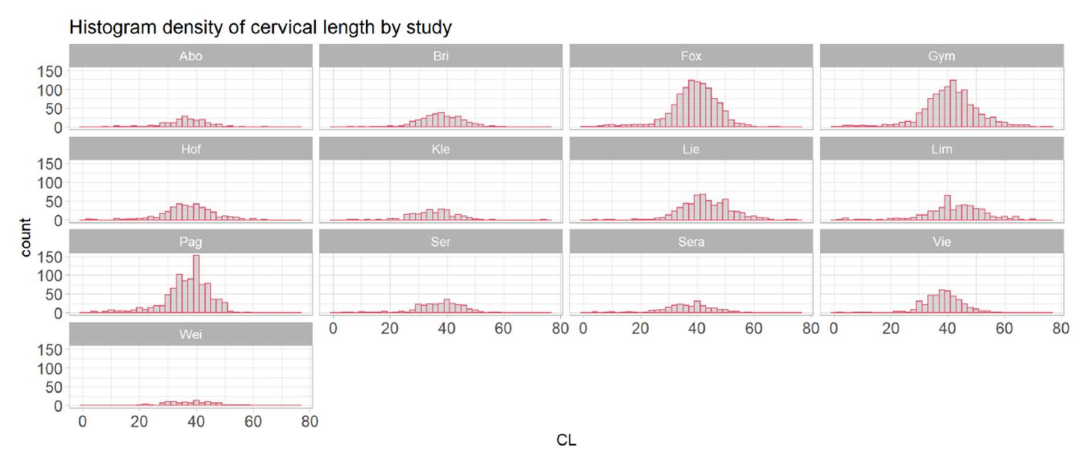

Supplement: online supplemental figure 1 [file bmjmed-4-1-s004.pdf]

Supplementary figure 2: scatterplot of CL by GA

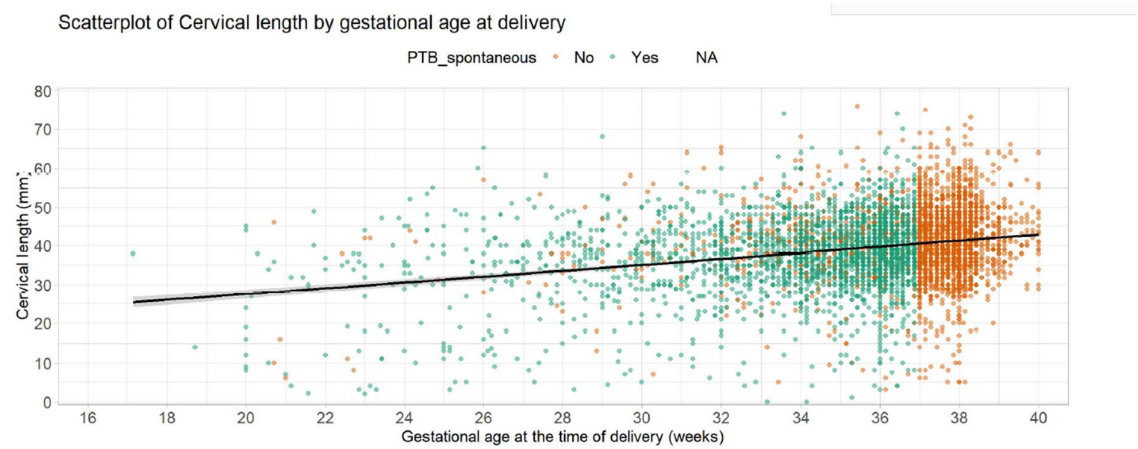

Supplement: online supplemental figure 2 [file bmjmed-4-1-s005.pdf]
