# Supplementary material for: Prognostic value of cervical length for spontaneous preterm birth in asymptomatic women with twin pregnancy: meta-analysis of individual participant data
Source: BMJ Med. 2025 Apr 16;4(1):e000877. doi: 10.1136/bmjmed-2024-000877 (PMC12056617; doi:10.1136/bmjmed-2024-000877)

Supplementary figure 3: additional models

Model 2: key demographic prognostic factors (maternal age, nulliparity)

Study	TE	SE	Weight	Hazard Ratio IV, Random, 95% CI
Ser	-0.061	0.0113	7.7%	0.941 [0.920; 0.962]
gym	-0.050	0.0063	18.3%	0.952 [0.940; 0.964]
sera	-0.045	0.0118	7.2%	0.956 [0.934; 0.979]
vie	-0.042	0.0137	5.5%	0.958 [0.933; 0.985]
Fox	-0.039	0.0059	19.8%	0.962 [0.951; 0.973]
Lim	-0.037	0.0069	16.3%	0.963 [0.950; 0.977]
Hof	-0.035	0.0128	6.2%	0.965 [0.941; 0.990]
Lie	-0.028	0.0070	16.0%	0.972 [0.959; 0.986]
wei	-0.018	0.0193	3.0%	0.983 [0.946; 1.020]
**Total (95% CI)**			**100.0%**	**0.960 [0.954; 0.967]**
**Prediction interval**				**[0.947; 0.974]**

Heterogeneity: $Tau^2 < 0.0001$; $Chi^2 = 10.43$, df = 8 (P = 0.24); $I^2 = 23.332\%$

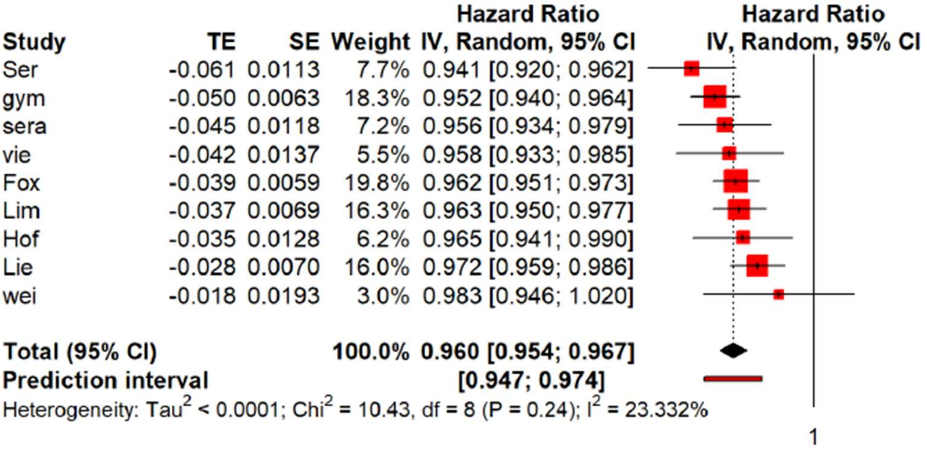

Model 3a: all potential prognostic factors

Study	TE	SE	Weight	Hazard Ratio IV, Random, 95% CI
Bri	-0.072	0.0116	7.1%	0.930 [0.909; 0.952]
Abo	-0.069	0.0123	6.8%	0.934 [0.911; 0.956]
Ser	-0.061	0.0113	7.3%	0.941 [0.920; 0.962]
gym	-0.050	0.0063	9.5%	0.952 [0.940; 0.964]
Hof	-0.045	0.0138	6.2%	0.956 [0.930; 0.982]
vie	-0.045	0.0142	6.1%	0.956 [0.930; 0.983]
sera	-0.045	0.0118	7.1%	0.956 [0.934; 0.979]
Fox	-0.039	0.0059	9.7%	0.962 [0.951; 0.973]
Lim	-0.037	0.0069	9.3%	0.963 [0.951; 0.977]
Lie	-0.025	0.0071	9.2%	0.975 [0.962; 0.989]
Kle	-0.020	0.0105	7.6%	0.980 [0.960; 1.000]
wei	-0.018	0.0193	4.4%	0.983 [0.946; 1.020]
pag	-0.013	0.0061	9.6%	0.987 [0.976; 0.999]
**Total (95% CI)**			**100.0%**	**0.960 [0.950; 0.970]**
**Prediction interval**				**[0.926; 0.995]**

Heterogeneity: $Tau^2 = 0.0002$; $Chi^2 = 47.86$, df = 12 (P < 0.01); $I^2 = 75\%$

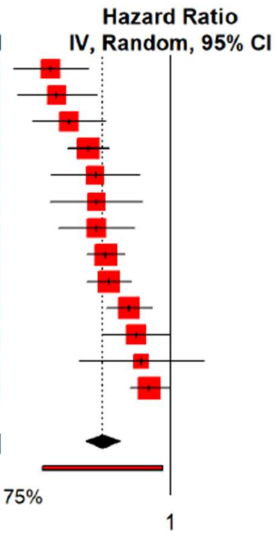

Model 3b: studies reporting all prognostic factors

Study	TE	SE	Weight	Hazard Ratio IV, Random, 95% CI
Ser	-0.061	0.0110	10.5%	0.941 [0.921; 0.961]
gym	-0.049	0.0060	19.9%	0.952 [0.941; 0.963]
vie	-0.045	0.0140	7.4%	0.956 [0.930; 0.983]
Hof	-0.045	0.0140	7.4%	0.956 [0.930; 0.983]
Fox	-0.039	0.0060	19.9%	0.962 [0.951; 0.973]
Lim	-0.037	0.0070	17.5%	0.964 [0.951; 0.977]
Lie	-0.025	0.0070	17.5%	0.975 [0.962; 0.989]
**Total (95% CI)**			**100.0%**	**0.959 [0.951; 0.968]**
**Prediction interval**				**[0.938; 0.981]**

Heterogeneity: $Tau^2 < 0.0001$; $Chi^2 = 10.90$, df = 6 ($P = 0.09$); $I^2 = 45\%$

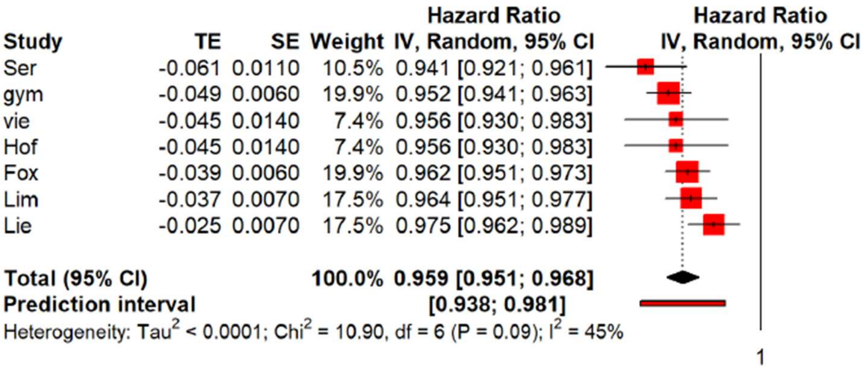

Supplement: online supplemental figure 3 [file bmjmed-4-1-s006.pdf]
