# Supplementary material for: Prognostic value of cervical length for spontaneous preterm birth in asymptomatic women with twin pregnancy: meta-analysis of individual participant data
Source: BMJ Med. 2025 Apr 16;4(1):e000877. doi: 10.1136/bmjmed-2024-000877 (PMC12056617; doi:10.1136/bmjmed-2024-000877)
Supplement: online supplemental table 1 [file bmjmed-4-1-s007.pdf]

**Supplementary Table 1:** excluded/unavailable studies

| Author | Year | Country | Number of participants | Reason |
|---|---|---|---|---|
| Sperling(1) | 2005 | Denmark | 383 | No response |
| Saccone(2) | 2019 | USA | 668 | Dataset missing |
| Qu(3) | 2011 | China | 427 | Unable to contact |
| El-Gharib(4) | 2017 | Egypt | 150 | No response |
| Ginsberg (5) | 2015 | Israel | 243 | No response |
| Janssen(6) | 2018 | USA | 359 | No response |
| Khalil(7) | 2013 | Saudi Arabia | 209 | No response |
| Pittini(8) | 2017 | Canada | 504 | Previously not consenting to data sharing |
| Dang(9) | 2018 | Vietnam | 150 | Unable to provide data within requested timeframe |
| Lynch(10) | 2020 | USA | 114 | Data sharing process not completed |

1. Sperling L, Kiil C, Larsen LU, Qvist I, Bach D, Wojdemann K, et al. How to identify twins at low risk of spontaneous preterm delivery. Ultrasound in Obstetrics & Gynecology. 2005;26(2):138-44.
2. Saccone G, Zullo F, Roman A, Ward A, Maruotti G, Martinelli P, et al. Risk of spontaneous preterm birth in IVF-conceived twin pregnancies. The journal of maternal-fetal & neonatal medicine : the official journal of the European Association of Perinatal Medicine, the Federation of Asia and Oceania Perinatal Societies, the International Society of Perinatal Obstet. 2019;32(3):369-76.
3. Qu SH, Shi CY, Chen Q, Chen JY, Sun WJ, Sun Y, et al. [Predictive value of cervical length by transvaginal sonography for preterm pregnancy during mid- and late-trimester of pregnancy]. Zhonghua fu chan ke za zhi. 2011;46(10):748-52.
4. El-Gharib MN, Albehoty SB. Transvaginal cervical length measurement at 22- to 26-week pregnancy in prediction of preterm births in twin pregnancies. The journal of maternal-fetal & neonatal medicine : the official journal of the European Association of Perinatal Medicine, the Federation of Asia and Oceania Perinatal Societies, the International Society of Perinatal Obstet. 2017;30(6):729-32.

5. Ginsberg Y, Goldstein I, Khatib N, Farago N, Weiner Z. Lower uterine segment thickness and cervical length measured between 16-25 weeks' gestation predict the etiology of preterm deliveries in twin gestations. American Journal of Obstetrics and Gynecology. 2015;212(1 SUPPL. 1):S134.
6. Janssen AE, Moore TA, Lovgren TR, Levine MG, Robertson AW, Dahlke JD. Cervical length surveillance frequency and the association of spontaneous preterm delivery in twin gestation. American Journal of Obstetrics and Gynecology. 2018;218(1 Supplement 1):S412-S3.
7. Khalil MI, Alzahrani MH, Ullah A. The use of cervical length and change in cervical length for prediction of spontaneous preterm birth in asymptomatic twin pregnancies. European Journal of Obstetrics & Gynecology and Reproductive Biology. 2013;169(2):193-6.
8. Pittini A, Barrett J, Stratulat V, Glanc P, Melamed N. Sonographic characteristics of the cervix in women with twin gestations at risk of preterm birth. American Journal of Obstetrics and Gynecology. 2017;216(1 Supplement 1):S171.
9. Dang VQ, Nguyen LK, He YTN, Vu KN, Phan MTN, Pham TD, et al. Cervical pessary versus vaginal progesterone for the prevention of preterm birth in women with a twin pregnancy and a cervix <38 mm: A randomized controlled trial. American Journal of Obstetrics and Gynecology. 2018;218(1 Supplement 1):S603-S4.
10. Lynch TA, Szlachetka K, Seligman NS. Second trimester uterocervical angle and spontaneous preterm birth in twins. The journal of maternal-fetal & neonatal medicine : the official journal of the European Association of Perinatal Medicine, the Federation of Asia and Oceania Perinatal Societies, the International Society of Perinatal Obstet. 2020;33(18):3125-31.
