# Supplementary material for: Prognostic value of cervical length for spontaneous preterm birth in asymptomatic women with twin pregnancy: meta-analysis of individual participant data
Source: BMJ Med. 2025 Apr 16;4(1):e000877. doi: 10.1136/bmjmed-2024-000877 (PMC12056617; doi:10.1136/bmjmed-2024-000877)
Supplement: online supplemental table 2 [file bmjmed-4-1-s008.pdf]

Supplementary Table 2 Sensitivity analyses

	Number of studies	Effect size	95% CI	Tau^2	I^2
Exclude women that receive treatment for preterm birth	13	HR: 0.961	0.952; 0.971	0.0002	67.9%
SPTB<37 weeks (binary outcome)	12	OR: 0.956	0.950; 0.963	<0.0001	13.3%
SPTB<34 weeks (binary outcome)	13	OR: 0.933	0.916; 0.950	0.0007	69.3%
SPTB<30 weeks (binary outcome)	13	OR: 0.902	0.875; 0.929	0.002	65.5%
Exclude studies in which any women received treatment	Not performed due to limited number of studies				
Exclude studies with an overall high risk of bias	Not performed due to limited number of studies				
