# Supplementary material for: Prognostic value of cervical length for spontaneous preterm birth in asymptomatic women with twin pregnancy: meta-analysis of individual participant data
Source: BMJ Med. 2025 Apr 16;4(1):e000877. doi: 10.1136/bmjmed-2024-000877 (PMC12056617; doi:10.1136/bmjmed-2024-000877)
Supplement: online supplemental table 3 [file bmjmed-4-1-s009.pdf]

Supplementary Table 3. Meta-analyses using different methods in the main model (model 1)

Outcomes	Inverse-variance method hazard ratio (95% confidence intervals)	REML-HKSJ method hazard ratio (95% confidence intervals)
SPTB<37 weeks	0.960 (0.951-0.969)	0.960 (0.950-0.970)
SPTB<34 weeks	0.932 (0.915-0.950)	0.932 (0.913-0.952)
