# Supplementary material for: Prognostic value of cervical length for spontaneous preterm birth in asymptomatic women with twin pregnancy: meta-analysis of individual participant data
Source: BMJ Med. 2025 Apr 16;4(1):e000877. doi: 10.1136/bmjmed-2024-000877 (PMC12056617; doi:10.1136/bmjmed-2024-000877)
Supplement: Supplementary file 10 [file bmjmed-2024-000877.reviewer_comments.pdf]

PEER REVIEW HISTORY

BMJ Medicine publishes all reviews undertaken for accepted manuscripts. Reviewers are asked to complete a checklist review form and are provided with free text boxes to elaborate on their assessment. These free text comments are reproduced below.

ARTICLE DETAILS

**TITLE (PROVISIONAL)**	Prognostic value of cervical length for spontaneous preterm birth in asymptomatic women with twin pregnancy: an individual participant data meta-analysis
**AUTHORS**	Hughes, Kelly; Aberoumand, Mason; Seidler, Anna; Swan, Phoebe; Aboulghar, Mona; de Lourdes Brizot, Maria; Brock, Clifton; Benito Vielba, Marta; Fox, N; Gyamfi-Bannerman, C; Kindinger, L; Pagani, G; Perales Marin, A; Seravalli, V; Di Tommaso, M; Weitzner, O; Worda, K; Staub, L; Brennecke, S; Thangaratinam, Shakila; Mol, Ben; Wang, Rui

VERSION 1 - REVIEW

**REVIEWER NAME**	Thornton, Jim
**REVIEWER AFFILIATION**	
**REVIEWER CONFLICT OF INTEREST**	None
**DATE REVIEW RETURNED**	19-Feb-2024

**GENERAL COMMENTS**	The topic is important and the methods appropriate.<br><br>The study was pre registered on PROSPERO and as far as I can see has followed the protocol.<br><br>IPD MA is the best way to combine studies like this.<br><br>I have a few minor issues.<br>1. The table on P 21 is not labelled. I assume it is table 1. included studies. It is difficult to check the source reference for these studies because the numeric ref is not included in the table. Also I can't see To in this table. This may be due to abbreviation problems. But there is no source with 1138 participants. Please clarify.<br><br>2. The table on p 23 is also not labelled.<br><br>3. In the methods the primary outcome is spontaneous PTB. The authors mention all PTB as a secondary outcome. However in the table on p 23 and in the figures the outcome is simply called PTB. Is this spontaneous or total? Please clarify.<br><br>4. In the SAP Gyamphi-Bannerman is abbreviated as Gya. But Gya does not appear in the tables or figs. I suspect she is Gym. Similarly where is To in the figures?<br><br>I think it would be clearer and less risky to give author names and main publication date in full in the results tables and figs<br><br>Overall a very nice paper. Recommend publication

REVIEWER NAME	Luo, Qiong
REVIEWER AFFILIATION	Women's Hospital, Zhejiang University School of Medicine, Obstetrics
REVIEWER CONFLICT OF INTEREST	None
DATE REVIEW RETURNED	01-May-2024

GENERAL COMMENTS	The manuscript is well-written, but I have some major concerns:<br>1. The last search was performed on 30/9/2020. However, there are many manuscripts research on predicting preterm birth in twin pregnancy, such as mid-trimester transvaginal sonographic cervical length in recent years, and it is recommended to re-searched and supplemented recently studies.<br>2.Limitation of this meta-analysis was the included studies were all retrospective cohort, which typically introduces selection bias from the outset,and it is adminted as ahthor, also author himself accepted.<br>3.In Figuar 3a ,I2 =74.492%, and in Figuar 3b ,I2 =69.267%. There were considerable heterogeneity, which was not explained by the authors.

REVIEWER NAME	Care, Angharad
REVIEWER AFFILIATION	University of Liverpool, Women's and Children's Health
REVIEWER CONFLICT OF INTEREST	None
DATE REVIEW RETURNED	07-Jun-2024

GENERAL COMMENTS	The aim of this IPD and meta-analysis was to quantify how well mid-trimester cervical length can predict spontaneous preterm birth in an asymptomatic twin population, using it as a prognostic rather than diagnostic factor.<br><br>Overall the study has shown as expected that increased cervical length confers a reduced risk of spontaneous preterm birth, and presents the associated hazard ratios. There has been a large amount of work that has gone into this study and the authors should be commended on obtaining such a large twin data cohort - a group that are frequently under studied. The study is unable to show a specific threshold at which sPTB would not occur, resulting in cervical length being a tool of limited clinical value in this population until there are proven thresholds for intervention / interventions. The authors have considered a clinical audience and estimated risks of spontaneous preterm birth at different gestational ages which has slightly more translational applicability.<br><br>My main issues with this paper are that it is very difficult to understand how this data is clinically applicable and feel like the authors could take this opportunity to try and explain the importance of this work. How would one apply a 1mm increase in hazard ratio in clinical practice? The table of probabilities is presented in probabilities rather than attempting to translate the language into a risk of e.g. 1 in 10 women to make the data more useful to a clinical audience.<br><br>I understand that you have used CL measurement as a continuous variable to 37 weeks to avoid data loss and potentially biased effect estimates, but was surprised in a twin study that the primary

	outcome of 37 weeks was chosen. Clinicians generally deliver MCDA twins at 36 weeks and DCDA twins at 37 weeks to avoid stillbirth, so they would really want to know what the risk of delivery is <34 weeks or <28 weeks when the burden of morbidity and mortality is greater and this information may influence clinical decision making - such as place of care.<br><br>Occasionally you refer to sPTB and in other places PTB - particularly in your tables. Could you please make it clear if you are referring to sPTB or ALL CAUSE PTB to avoid confusion<br><br>Could you please define spontaneous preterm birth for this study? i.e. Did you include all iatrogenic deliveries for PPROM as a spontaneous preterm birth event?<br><br>It would be useful to have more information around the studies and populations included in your IPD. Can you explain the variation in rates of PTB <37 weeks from 10% to 74% in studies in your cohort?<br><br>It would be useful to have some demographic data from women included in the IPD in your appendices, I appreciate that in each model the numbers are different based on differences in available data.<br><br>The last search was performed on 30th September 2020 - this is nearly 4 years ago now, is it possible for the search to be updated? I note the difficulties with data sharing you very eloquently explained and thank you for highlighting these important issues.<br><br>Page 12, line 27 - Results. 3885 abstracts underwent screening. The flow diagram states 3884 underwent screening. Please correct typo.

**REVIEWER NAME**	Riley, Richard
**REVIEWER AFFILIATION**	University of Birmingham, Institute of Applied Health Research
**REVIEWER CONFLICT OF INTEREST**	I receive royalties for my IPD mea-analysis book.<br>I have worked with a few of the authors on previous IPD meta-analysis projects.
**DATE REVIEW RETURNED**	08-Jul-2024

**GENERAL COMMENTS**	Thank you for the opportunity to review this IPD meta-analysis submission to BMJ Medicine. The article is an IPD meta-analysis to examine whether cervical length has (adds) prognostic value for the outcome of spontaneous preterm birth. Without IPD, meta-analysis of prognostic factor studies is very problematic due to different analysis approaches (e.g. categorisation thresholds, adjustment factors, etc) – and so the IPD project here as strong rationale to help resolve an ongoing debate in the field about whether cervical length is a prognostic factor.<br><br>The IPD meta-analysis project is very well done. Although the last search for relevant studies was done in Sept 2020, the process of obtaining and cleaning etc IPD takes many years, so the article should not be penalised for this. I like the clear PICOTS system description. It is also important to see that cervical length is modelled on its continuous scale and not categorised, and clear strategy for handled other prognostic variables is made by fitting different models with different sets of adjustment terms. It is also

	good to see that the findings show a clear prognostic value of cervical length regardless, and with no evidence of heterogeneity.<br><br>I have some comments which the authors should consider for a revision moving forwards<br><br>1) The inclusion criteria was studies that previously had assessed the association between cervical length (CL) and preterm birth ('… were searched to identify eligible studies examining the association…'). I wonder, does this therefore exclude other high-quality datasets that may have this information (e.g. in a routine database)? In other words, if a study had to have already looked at this association, for IPD to be sought, is there a concern that the IPD collected is a 'biased' representation of all the evidence available? This is just for discussion and for the authors to clarify better in the article.<br>2) I think the use of the word 'predict' is confusing (eg, in the title). Indeed the authors interchange between association, prognostic value and predict etc – I think they should be more consistent to use association or prognostic value, and refer to this as a prognostic factor study (which to be fair they do for most parts). The word 'predict' implies (to me) that they are using CL to predict the absolute risk of the outcome, but this is not the focus here – as there is no examination of predictive performance in terms of measures such as calibration, discrimination and clinical utility. Therefore, revise the title and be careful with the word predict elsewhere in the article.<br>3) 'Where studies also reported medically indicated preterm birth, these were treated as censored observations.' – this creates an artificial world where no medically indicated preterm births can happen. I think this is in principle correct, as the hazard ratios then tell us the effect of CL on the risk of spontaneous preterm birth. But I am worried about informative censoring – those babies that have a medically indicated preterm birth are (I assume) more likely to have had a spontaneous preterm birth too. So, by simply censoring individuals who had a medically indicated preterm birth, will this not make the actual risk of spontaneous preterm birth too low? So does this make the study's interpretation to be about the prognostic effect of CL on spontaneous preterm births for babies that would not ever receive medically induced preterm birth during the 37 weeks of pregnancy? I think the exact interpretation of all this needs to be better explained for the reader.<br>4) ROB assessment using QUIPDS is appropriate but: 'For domains on adjustment for other prognostic factors and statistical analysis and reporting, the assessments were based on the methods used in the published study' – given that the authors have the IPD, why assess a study's RoB based on their published study analyses, and not just on the IPD itself (as the authors will analyse the IPD themselves)<br>5) Models 3 and 4 are the same model, but differ by the IPD included, so to call them model 3 and 4 is misleading. Better to stick to model 3 in both situations, but then present results a few times for model 3 conditional on the IPD included.<br>6) Indeed, it is not clear how missing predictors were handled in the models 1 or 2 either<br>7) 'the proportion of heterogeneity due to between-study variance was determined using I2 statistic' – the authors mean 'the percentage of the total variability in study estimates due to between-study variance was determined using I2 statistic'<br>8) Methods say prediction interval was calculated, but does not say

	if 95% and how it was calculated<br>9) ‘Small study effect was investigated …’ – small study effectS<br>10) ‘Publication bias was explored by producing a contour-enhanced funnel plot’ – the authors mean small study effects was explored, as publication bias is only one causes of asymmetric funnel plot<br>11) The two-stage approach should be justified better: were most studies in the meta-analysis not small? If so, then this is sensible, but if not then I worry a two-stage approach is a poor approximation (in the second stage).<br>12) For the random effects MA model, what approach was used for estimation? And for derivation of 95% CIs? Was the Hartung-Knapp approach used?<br>13) ‘In addition, the non-linear association between cervical length and preterm birth were investigated with generalised additive models using cubic splines’ – right at the end of the methods, the authors now refer very briefly to this (in my opinion) most insightful analysis that allows non-linear associations to be considered. Indeed, it is a one-stage analysis – so different to the two-stage approach previously detailed – and so we need details on how clustering was handled, how adjustment for other prognostic factors was handled, (e.g. ignored study clustering, stratified the intercept / placed random effect on intercept, stratified the adjustment prognostic factor terms, etc) and estimation methods. ML estimation is downwardly biased, but centering of covariates can help to improve tau2 estimate.<br>14) In terms of the splines, how were knot locations identified and were these forced to be the same in each study? Please give details.<br>15) Was the proportional hazards assumption assessed within-study for the two-stage IPDMA? E.g. was HR really a constant over time? Seems strong assumption as CL may make more impact earlier (nearer measurement time) than later?<br>16) How were the adjustment (existing prognostic factor) variables identified? Based on their prognostic value in the literature?<br>17) ‘The number of participants in one dataset did not match the publication (there were 29 fewer pregnancies in the dataset compared to the publication described),(37) and a query about this to the author did not receive a response.’ – what are the consequences of this? Was it excluded / deemed high(er) risk of bias?<br>18) GA at CL measurement can vary between 16 and 24 weeks (see PICOTS): thus the startpoint (moment for prognostication) varies across studies – therefore, I was surprised to not see a sensitivity analysis where studies are only combined if GA was very similar (e.g. 19-21 weeks). Could this be added – regardless, the interpretation of the summary results needs careful interpretation of the GA varies by 2 months.<br>19) QUIPS was used, which I think is correct, but the ‘confounding domain’ is not appropriate language, and should be rather termed the ‘adjustment for other prognostic factors’ (https://www.bmj.com/content/364/bmj.k4597) domain. Confounding is strictly a causal aspect which does not work well here.<br>20) ‘There was no evidence to suggest a non-linear relationship between cervical length and GA at birth’ – no results given, which is a missed opportunity. Can we have a graph please with a 95% CI for the non-linear association<br>21) In the methods, make sure to say that CL was modelled as continuous and a linear association on the log-hazard scale was assumed<br>22) The results are projected back onto the absolue risk scale, but

	there are no details on how this was done. In particular, although there is no evidence of heterogeneity of the HR, there will be heterogeneity in baseline risk across studies and countries, so it is not clear what the absolute risk values refer to exactly. Also, when doing this, the absolute risks are presented by CL value, but it is not clear how other adjustment variables (prognostic factors) were accounted for. Are they integrated over, in some sense? Otherwise the authors are taking the HRs and ignoring the adjustment variables (Table 2 fallacy)<br>23) I think the table of predicted risks should be taken out, for the reasons made in the point above, but also because there is no assessment of calibration of these risks. As mentioned earlier, the study focuses on prognostic value and HRs, but this move at the end to risk prediction requires different methodology for developing models (e.g. penalisation and shrinkage) and evaluating (e.g. calibration checks) them. That could be for a different paper.<br>24) 'It showed a 6.7% reduction in risk of SPTB prior to 34 weeks' gestation for every millimetre increase in cervical length, and a 3.8% reduction in risk of SPTB less than 37 weeks.' – careful. It is not a 6.7% or 3.8% reduction in RISK but (I think) RATE (hazard rate), that the authors have identified. Please check.<br>25) Self-indulgent note: the ref 7 is missing the book editor surnames.<br><br>I hope these comments are useful for the authors moving forwards.

VERSION 1 – AUTHOR RESPONSE

Reviewer 1

Reviewer 1 comment 1 The table on P 21 is not labelled. I assume it is table 1. included studies. It is difficult to check the source reference for these studies because the numeric ref is not included in the table. Also I can't see To in this table. This may be due to abbreviation problems. But there is no source with 1138 participants. Please clarify.

Response Apologies for the discrepancies here. Table 1's label was underneath the table, but it now precedes the table which we hope improves its prominence.

References had been omitted from table as they would not correspond with reference numbering in the main paper as the files are separate. They are now added, and we anticipate they can be combined at the time of editing.

The paper by To et al 2006 was excluded due to the data collection period commencing before 2000, in accordance with our listed exclusion criteria. It should have been removed from the SAP.

Changes made Table title moved. To et al removed from SAP.

Location of changes Table 1

Reviewer 1 comment 2 The table on p 23 is also not labelled.

Response A label has been added

Changes made A label has been added

Location of changes Table 2

Reviewer 1 comment 3 In the methods the primary outcome is spontaneous PTB. The authors mention all PTB as a secondary outcome. However in the table on p 23 and in the figures the outcome is simply called PTB. Is this spontaneous or total? Please clarify.

Response Thank you for this observation. The figures should indicate spontaneous preterm birth.

Changes made Figure titles and headings updated accordingly.

Location of changes Table 1, 2, Figure 3a, 3b, 4a

Reviewer 1 comment 4 In the SAP Gyamphi-Bannerman is abbreviated as Gya. But Gya does not appear in the tables or figs. I suspect she is Gym. Similarly where is To in the figures?
Response Gyamfi-Bannerman was the supervising author for the Brock and Moroz papers (as specified in Figure 2), and our initial point of contact with that group of researchers. The data was received as a single dataset, and is referred to as “gym” in the output.
The paper by To et al 2006 was excluded as mentioned in our response to comment #1.

Changes made Clarifying comment added in results section.
Location of changes P10

Reviewer 2
Reviewer 2 comment 1 The last search was performed on 30/9/2020. However, there are many manuscripts research on predicting preterm birth in twin pregnancy, such as mid-trimester transvaginal sonographic cervical length in recent years, and it is recommended to re-searched and supplemented recently studies.
Response Unfortunately, this has been resulted from a combination of extremely slow process of data sharing (2.5+ years) and a delay in manuscript handling process within the journal (9+ months). We would very much like to have a more up-to-date search also, however, as we have described, and Professor Riley (Reviewer 4) has acknowledged, the process of data sharing is extremely slow and datasets cannot remain open indefinitely.
Changes made n/a
Location of changes n/a

Reviewer 2 comment 2 Limitation of this meta-analysis was the included studies were all retrospective cohort, which typically introduces selection bias from the outset,and it is adminted as ahthor, also author himself accepted.
Response As the reviewer states, we have acknowledged the shortcomings in the primary literature. This study design cannot overcome these, but it is the best treatment of the available data.
Changes made n/a
Location of changes n/a

Reviewer 2 comment 3 In Figuar 3a ,I2 =74.492%, and in Figuar 3b ,I2 =69.267%. There were considerable heterogeneity, which was not explained by the authors.
Response We have explained in the results section that between-study heterogeneity was low (this is measured by tau2). As Professor Riley also mentions, rather famously, “I2 measures proportion of total variability due to between-study heterogeneity”, rather than quantifying between-study heterogeneity.
Changes made I2 now reported.
Location of changes P11

Reviewer 3
Reviewer 3 comment 1 My main issues with this paper are that it is very difficult to understand how this data is clinically applicable and feel like the authors could take this opportunity to try and explain the importance of this work. How would one apply a 1mm increase in hazard ratio in clinical practice? The table of probabilities is presented in probabilities rather than attempting to translate

the language into a risk of e.g. 1 in 10 women to make the data more useful to a clinical audience.
Response We appreciate this perspective, but also acknowledge the alteration in statistical approach required. The opposing perspective is provided by Reviewer 4 below.
Changes made Additional rows added to probabilities table to express these in terms of risk.
Location of changes Supplemental Table 2

Reviewer 3 comment 2 I understand that you have used CL measurement as a continuous variable to 37 weeks to avoid data loss and potentially biased effect estimates, but was surprised in a twin study that the primary outcome of 37 weeks was chosen. Clinicians generally deliver MCDA twins at 36 weeks and DCDA twins at 37 weeks to avoid stillbirth, so they would really want to know what the risk of delivery is <34 weeks or <28 weeks when the burden of morbidity and mortality is greater and this information may influence clinical decision making - such as place of care.
Response We have utilised 37 weeks' gestation as a cutoff because it is the standard definition of PTB whether singleton or multiple, however we have also presented data for spontaneous PTB <34 weeks (and <28 weeks, within supplementary figure 3). We acknowledge in our discussion that most data were collected in women with DCDA twin pregnancies, so we have recommended that our conclusions are applicable in this population. Given that NICE recommends birth at 37-38 weeks' gestation for uncomplicated DCDA twin pregnancies,(1) we contend that 37 weeks is still a clinically relevant figure.
Changes made Additional statement added in discussion section
Location of changes P12

Reviewer 3 comment 3 Occasionally you refer to sPTB and in other places PTB - particularly in your tables. Could you please make it clear if you are referring to sPTB or ALL CAUSE PTB to avoid confusion
Response We have reviewed all instances of these terms in the documents.
Changes made Table 2a & b file name updated. Occasional substitution of "SPTB" for "spontaneous preterm birth".
Location of changes Table 2a & b file name.

Reviewer 3 comment 4 Could you please define spontaneous preterm birth for this study? i.e. Did you include all iatrogenic deliveries for PPROM as a spontaneous preterm birth event?
Response Yes, PPROM was counted as a spontaneous preterm birth.
Changes made Additional sentence to clarify definitions.
Location of changes P8

Reviewer 3 comment 5 It would be useful to have more information around the studies and populations included in your IPD. Can you explain the variation in rates of PTB <37 weeks from 10% to 74% in studies in your cohort?
Response Thank you for this very worthwhile consideration.
Changes made Additional paragraph added to discussion section to acknowledge and explore this variation.
Location of changes P13

Reviewer 3 comment 6 It would be useful to have some demographic data from women included in the IPD in your appendices, I appreciate that in each model the numbers are different based on differences in available data.
Response Unfortunately, we do not have detailed demographic information across all studies, but we have listed the demographic data available in the included studies. We have also described the

limitations of the datasets in our analysis.
Changes made We have listed the demographic data available in the included studies
Location of changes Table 1

Reviewer 3 comment 7 The last search was performed on 30th September 2020 - this is nearly 4 years ago now, is it possible for the search to be updated? I note the difficulties with data sharing you very eloquently explained and thank you for highlighting these important issues.
Response See also our response to Reviewer 2, comment 1.
Changes made n/a
Location of changes n/a

Reviewer 3 comment 8 Page 12, line 27 - Results. 3885 abstracts underwent screening. The flow diagram states 3884 underwent screening. Please correct typo.
Response Thank you for detecting this.
Changes made Number corrected in Results section.
Location of changes P10

Reviewer 4
Reviewer 4 comment 1 The inclusion criteria was studies that previously had assessed the association between cervical length (CL) and preterm birth ('… were searched to identify eligible studies examining the association…'). I wonder, does this therefore exclude other high-quality datasets that may have this information (e.g. in a routine database)? In other words, if a study had to have already looked at this association, for IPD to be sought, is there a concern that the IPD collected is a 'biased' representation of all the evidence available? This is just for discussion and for the authors to clarify better in the article.
Response Earlier in the manuscript we describe our eligibility criteria, which includes studies whose primary purpose was not to evaluate a relationship between cervical length and spontaneous preterm birth, but report these variables as part of another research question (e.g. treatment trials). We agree that this could be more accurately described in the methods section.
Changes made Sentence amended to read, "to identify eligible studies reporting both mid-trimester transvaginal sonographic cervical length and gestational age at birth".
Location of changes P4 Methods section

Reviewer 4 comment 2 I think the use of the word 'predict' is confusing (eg, in the title). Indeed, the authors interchange between association, prognostic value and predict etc – I think they should be more consistent to use association or prognostic value, and refer to this as a prognostic factor study (which to be fair they do for most parts). The word 'predict' implies (to me) that they are using CL to predict the absolute risk of the outcome, but this is not the focus here – as there is no examination of predictive performance in terms of measures such as calibration, discrimination and clinical utility. Therefore, revise the title and be careful with the word predict elsewhere in the article.
Response We appreciate the distinction and have made the recommended changes – using "prognostic value" across the paper.
Changes made Revised title and terminology.
Location of changes Various.

Reviewer 4 comment 3 'Where studies also reported medically indicated preterm birth, these were treated as censored observations.' – this creates an artificial world where no medically indicated

preterm births can happen. I think this is in principle correct, as the hazard ratios then tell us the effect of CL on the risk of spontaneous preterm birth. But I am worried about informative censoring – those babies that have a medically indicated preterm birth are (I assume) more likely to have had a spontaneous preterm birth too. So, by simply censoring individuals who had a medically indicated preterm birth, will this not make the actual risk of spontaneous preterm birth too low? So does this make the study's interpretation to be about the prognostic effect of CL on spontaneous preterm births for babies that would not ever receive medically induced preterm birth during the 37 weeks of pregnancy? I think the exact interpretation of all this needs to be better explained for the reader.
Response Approximately half of all preterm births are iatrogenic. It is expected that pathologies that necessitate iatrogenic preterm birth occur independently of cervical length. We have used "any preterm birth" (both iatrogenic and spontaneous preterm birth), as a secondary outcome to help exclude an unobserved association between cervical length and iatrogenic preterm birth. We reported the findings of both outcomes in the results section. We saw similar prognostic value of cervical length on both outcomes.
Changes made Further statement to clarify
Location of changes P14

Reviewer 4 comment 4 ROB assessment using QUIPDS is appropriate but: 'For domains on adjustment for other prognostic factors and statistical analysis and reporting, the assessments were based on the methods used in the published study' – given that the authors have the IPD, why assess a study's RoB based on their published study analyses, and not just on the IPD itself (as the authors will analyse the IPD themselves)
Response This was incorrectly reported, and was a phrase from a very early iteration of the manuscript. The RoB assessment was based on the IPD itself. We thank Prof Riley for alerting us to our error.
Changes made Sentence amended in "Risk of bias in individual studies" section
Location of changes P8

Reviewer 4 comment 5 Models 3 and 4 are the same model, but differ by the IPD included, so to call them model 3 and 4 is misleading. Better to stick to model 3 in both situations, but then present results a few times for model 3 conditional on the IPD included.
Response We have made the requested changes.
Changes made Now referred to Model 3a and 3b in manuscript and Supplemental Figure 3
Location of changes P9, Supplementary Figure 3

Reviewer 4 comment 6 Indeed, it is not clear how missing predictors were handled in the models 1 or 2 either
Response We used complete case analysis and have stated this in the methods section
Changes made Additional statement in methods section
Location of changes P9

Reviewer 4 comment 7 'the proportion of heterogeneity due to between-study variance was determined using I2 statistic' – the authors mean 'the percentage of the total variability in study estimates due to between-study variance was determined using I2 statistic'
Response Thank you, amended as suggested.
Changes made As above.
Location of changes P9

Reviewer 4 comment 8 Methods say prediction interval was calculated, but does not say if 95% and how it was calculated
Response We have clarified that it is the 95% prediction interval based on the inverse-variance method.
Changes made Sentence amended.
Location of changes Methods section p10

Reviewer 4 comment 9 ‘Small study effect was investigated …’ – small study effectS
Response Thank you, we have corrected this accordingly.
Changes made As above
Location of changes P9

Reviewer 4 comment 10 ‘Publication bias was explored by producing a contour-enhanced funnel plot’ – the authors mean small study effects was explored, as publication bias is only one causes of asymmetric funnel plot
Response Thank you for highlighting this distinction. We have corrected this.
Changes made This sentence has been updated, and the first author has learned some more about this topic.
Location of changes P9

Reviewer 4 comment 11 The two-stage approach should be justified better: were most studies in the meta-analysis not small? If so, then this is sensible, but if not then I worry a two-stage approach is a poor approximation (in the second stage).
Response Thank you. Most studies are not small and therefore two-stage is justified. We have added this in the methods section.
Changes made Two-stage approach was chosen as the size of most included studies was not small.
Location of changes P10

Reviewer 4 comment 12 For the random effects MA model, what approach was used for estimation? And for derivation of 95% CIs? Was the Hartung-Knapp approach used?
Response We used the inverse-variance method as specified in the SAP. We acknowledge that now it is more recommended to use REML for estimation and HKSJ approach for derivation of 95% CI. In this case, it does not change the findings or interpretation much. We acknowledge this in the discussion section.
Changes made Added a sentence on MA model in the methods and added a limitation in the discussion
Location of changes P9 &14

Reviewer 4 comment 13 ‘In addition, the non-linear association between cervical length and preterm birth were investigated with generalised additive models using cubic splines’ – right at the end of the methods, the authors now refer very briefly to this (in my opinion) most insightful analysis that allows non-linear associations to be considered. Indeed, it is a one-stage analysis – so different to the two-stage approach previously detailed – and so we need details on how clustering was handled, how adjustment for other prognostic factors was handled, (e.g. ignored study clustering, stratified the intercept / placed random effect on intercept, stratified the adjustment prognostic factor terms, etc) and estimation methods. ML estimation is downwardly biased, but centering of covariates can help to improve tau2 estimate.

Response The non-linear association was still investigated in a two-stage model – for details please refer to the response to the comment 14 and 20 below.
Changes made n/a
Location of changes n/a

Reviewer 4 comment 14 In terms of the splines, how were knot locations identified and were these forced to be the same in each study? Please give details.
Response Four knots were used, the number and position were determined as recommended by in the IPD meta-analysis Handbook.
Changes made Additional sentence in methods section
Location of changes P11

Reviewer 4 comment 15 Was the proportional hazards assumption assessed within-study for the two-stage IPDMA? E.g. was HR really a constant over time? Seems strong assumption as CL may make more impact earlier (nearer measurement time) than later?
Response Yes – this is indeed a strong assumption, and CL may indeed have more impact earlier as indicated in HR for SPTB<34 weeks. That is the reason why we also presented SPTB as a binary outcome in a series of sensitivity analyses (SPTB <37 weeks, <34 weeks and <30 weeks, respectively). As a binary outcome, such a strong assumption is not needed.
Time-to-event outcome and binary outcome can supplement each other, demonstrating the validity of the prognostic value of cervical length. We have added some explanation in the text to reflect this.
Changes made Additional explanatory sentence in discussion
Location of changes P14

Reviewer 4 comment 16 How were the adjustment (existing prognostic factor) variables identified? Based on their prognostic value in the literature?
Response Existing prognostic factors for SPTB are well-described in the literature and commonly (but unfortunately, as we have observed, not consistently!) collected in this area of clinical research. They often feature in clinical practice guidelines. (2)
We have reflected this in the methods section.
Changes made n/a
Location of changes n/a

Reviewer 4 comment 17 'The number of participants in one dataset did not match the publication (there were 29 fewer pregnancies in the dataset compared to the publication described),(37) and a query about this to the author did not receive a response.' – what are the consequences of this? Was it excluded / deemed high(er) risk of bias?
Response Apologies, this was from an earlier version of the draft and this issue has been fixed – with consistent number of pregnancies included in the dataset and the publication.
Changes made This sentence was removed.
Location of changes P10

Reviewer 4 comment 18 GA at CL measurement can vary between 16 and 24 weeks (see PICOTS): thus the startpoint (moment for prognostication) varies across studies – therefore, I was

surprised to not see a sensitivity analysis where studies are only combined if GA was very similar (e.g. 19-21 weeks). Could this be added – regardless, the interpretation of the summary results needs careful interpretation of the GA varies by 2 months.
Response We agree! We state in “Data items” our treatment of this issue: “In cases where multiple CL measurements were available per participant, we selected the measurement closest to 20 weeks gestation due to it coinciding with the usual morphology ultrasound.” (p7). We have had to leave the measurements taken at other points during the period of interest to a separate project.

Changes made n/a
Location of changes n/a

Reviewer 4 comment 19 QUIPS was used, which I think is correct, but the ‘confounding domain’ is not appropriate language, and should be rather termed the ‘adjustment for other prognostic factors’ (https://www.bmj.com/content/364/bmj.k4597) domain. Confounding is strictly a causal aspect which does not work well here.
Response This has been adjusted as advised.
Changes made Domain name corrected.
Location of changes P11

Reviewer 4 comment 20 ‘There was no evidence to suggest a non-linear relationship between cervical length and GA at birth’ – no results given, which is a missed opportunity. Can we have a graph please with a 95% CI for the non-linear association
Response Thank you for your interest in this part of the analysis. We have provided a graph as requested.
Changes made Additional figure referred to from the text, and uploaded.
Location of changes Figure 5

Reviewer 4 comment 21 In the methods, make sure to say that CL was modelled as continuous and a linear association on the log-hazard scale was assumed
Response We have added this as suggested.
Changes made Additional information added.
Location of changes P9

Reviewer 4 comment 22 The results are projected back onto the absolute risk scale, but there are no details on how this was done. In particular, although there is no evidence of heterogeneity of the HR, there will be heterogeneity in baseline risk across studies and countries, so it is not clear what the absolute risk values refer to exactly. Also, when doing this, the absolute risks are presented by CL value, but it is not clear how other adjustment variables (prognostic factors) were accounted for. Are they integrated over, in some sense? Otherwise the authors are taking the HRs and ignoring the adjustment variables (Table 2 fallacy)
Response We have added some further detail in the methods section. The results are based on the primary model which only includes two variables (cervical length and gestational weeks at measurement, the latter of which was a gestational weeks closest to 20 weeks). Therefore we believe adjustment variables should not be of concern here.
Changes made Additional sentence in methods section
Location of changes P10
Reviewer 4 comment 23 I think the table of predicted risks should be taken out, for the reasons made in the point above, but also because there is no assessment of calibration of these risks. As

mentioned earlier, the study focuses on prognostic value and HRs, but this move at the end to risk prediction requires different methodology for developing models (e.g. penalisation and shrinkage) and evaluating (e.g. calibration checks) them. That could be for a different paper.
Response We are grateful for the perspective here. Meanwhile we had clinicians' feedback (e.g. Reviewer 3 comment 1, but also direct discussions with clinician colleagues) requesting clinically relevant information that can be used for counselling patients. While we have attempted to treat the data appropriately, most obstetricians will find it extremely difficult to use the hazard ratios with continuous cervical length to assist with clinical counselling. We fully acknowledge that the predicted risks part warranties a different paper including model development and evaluations. Given that this table is based on model 1 consisting of two variables only, we wonder whether a compromised approach - moving the table to the supplemental materials – is acceptable at this stage. If not, we are open to the request to remove the table completely.
Changes made Table of predicted risks moved to supplemental table.
Location of changes Supplemental table 2

Reviewer 4 comment 24 'It showed a 6.7% reduction in risk of SPTB prior to 34 weeks' gestation for every millimetre increase in cervical length, and a 3.8% reduction in risk of SPTB less than 37 weeks.' – careful. It is not a 6.7% or 3.8% reduction in RISK but (I think) RATE (hazard rate), that the authors have identified. Please check.
Response Thank you, we have checked and corrected this in the abstract and manuscript.
Changes made "Rate" substituted.
Location of changes P4, P11, P15

Reviewer 4 comment 25 Self-indulgent note: the ref 7 is missing the book editor surnames.
Response Apologies, we incorrectly expected that the reference manager would display the editors' names in the reference.
Changes made All book authors added to the reference
Location of changes Reference list

1. Twin and triplet pregnancies: NICE guideline2024 09/12/2024. Available from: https://www.nice.org.uk/guidance/ng137/resources/twin-and-triplet-pregnancy-pdf-66141724389829.
2. Sentilhes L, Senat MV, Ancel PY, Azria E, Benoist G, Blanc J, et al. Prevention of spontaneous preterm birth: Guidelines for clinical practice from the French College of Gynaecologists and Obstetricians (CNGOF). European journal of obstetrics, gynecology, and reproductive biology. 2017;210:217-24.

VERSION 2 – REVIEW

**REVIEWER NAME**	Riley, Richard
**REVIEWER AFFILIATION**	University of Birmingham, Institute of Applied Health Research
**REVIEWER CONFLICT OF INTEREST**	
**DATE REVIEW RETURNED**	20-Jan-2025

**GENERAL COMMENTS**	This IPD meta-analysis continues to be an immense undertaking and I am very grateful for the authors replying to my comments and suggestions in such detail and honesty, and addressing the points made generally well. I only have a few (mostly minor) suggestions remaining:<br>1) Complete case analyses was done – could this be mentioned as a limitation in the discussion? Dealing with missing predictors in IPDMA is a very tricky problem, and the issue of different variables being recorded by different studies is challenging, that I understand the authors focus on a complete case analysis here; in further work using this database, accounting for missing data would be welcome.<br>2) I still think the cumulative risks would be better avoided entirely, not just placed in the supplementary material, or at least presented in the supp material with huge notes of caution when referred to in the main text. I appreciate the authors' state that the clinicians they spoke with want them, but they surely want the most reliable ones based on the right methodology. For this reason, I disagree with the statement 'we have derived a table of clinically relevant figures which may assist in SPTB risk assessment for asymptomatic women' – I think this is too premature and should be considered with proper prediction modelling methodology for risk prediction, in a follow-up study.<br>3) The authors now add that 'Two-stage approach was selected because most included studies were of sufficient size' – suggest 'Two-stage approach was selected because most included studies were of sufficient size for the assumptions to be appropriate'.<br>4) Please add to Table 1 the number of events per study (not just the %s) and also add all the abbreviations underneath (i.e. for CL, SPTB)<br>5) cervical length is not consistently abbreviated (e.g. see paragraph 2 of 'study characteristics', and first line of page 13).<br>6) '… more accepted to use restricted maximum likelihood for estimation and Hartung-Knapp-Sidik-Jonkman approach, however in this case the findings and interpretation are similar' – add reference to any results to support this in the supp material?<br>7) Figure 4: add a note to say that the y-axis is on the log-scale.<br>I look forward to see the paper published in due course, and congratulate the authors again on their hard work to produce this excellent article.

VERSION 2 – AUTHOR RESPONSE

1) Complete case analyses was done – could this be mentioned as a limitation in the discussion? Dealing with missing predictors in IPDMA is a very tricky problem, and the issue of different variables being recorded by different studies is challenging, that I understand the authors focus on a complete case analysis here; in further work using this database, accounting for missing data would be welcome.

We have added a comment and reference on complete case analysis as a potential limitation (p 15/16, ref #56)

2) I still think the cumulative risks would be better avoided entirely, not just placed in the supplementary material, or at least presented in the supp material with huge notes of caution when referred to in the main text. I appreciate the authors' state that the clinicians they spoke with want them, but they surely want the most reliable ones based on the right methodology. For this reason, I disagree with the statement 'we have derived a table of clinically relevant figures which may assist in SPTB risk assessment for asymptomatic women' – I think this is too premature and should be considered with proper prediction modelling methodology for risk prediction, in a follow-up study.

We have deleted the table and references to the table of cumulative risks

3) The authors now add that 'Two-stage approach was selected because most included studies were of sufficient size' – suggest 'Two-stage approach was selected because most included studies were of sufficient size for the assumptions to be appropriate'.

We have amended the sentence as suggested

4) Please add to Table 1 the number of events per study (not just the %s) and also add all the abbreviations underneath (i.e. for CL, SPTB)

We have added the number of events per study to Table 1 as requested.

5) cervical length is not consistently abbreviated (e.g. see paragraph 2 of 'study characteristics', and first line of page 13).

We have, at times, left "cervical length" typed in full for emphasis or ease of reading, but are happy to review these with the editorial team if our intent is not achieved. We have altered a few instances to the abbreviation where we felt it did not adversely impact reading fluency.

6) '… more accepted to use restricted maximum likelihood for estimation and Hartung-Knapp-Sidik-Jonkman approach, however in this case the findings and interpretation are similar' – add reference to any results to support this in the supp material?

We include a supplementary table (Supplementary Table 3) demonstrating the comparable results using REML & HSKJ. Differences only affected the third decimal place.

7) Figure 4: add a note to say that the y-axis is on the log-scale.

Figure 4) now has the y-axis labelled as on the log-scale
